# PAF1 cooperates with YAP1 in metaplastic ducts to promote pancreatic cancer

**DOI:** 10.1038/s41419-022-05258-x

**Published:** 2022-10-01

**Authors:** Rama Krishna Nimmakayala, Ayoola O. Ogunleye, Seema Parte, Nivedeta Krishna Kumar, Pratima Raut, Venkatesh Varadharaj, Naveen Kumar Perumal, Palanisamy Nallasamy, Sanchita Rauth, Jesse L. Cox, Subodh M. Lele, Surinder K. Batra, Moorthy P. Ponnusamy

**Affiliations:** 1 Department of Biochemistry and Molecular Biology, College of Medicine, University of Nebraska Medical Center, Omaha, NE 68198-5870 USA; 2 Department of Pathology and Microbiology, College of Medicine, University of Nebraska Medical Center, Omaha, NE USA; 3 Eppley Institute for Research in Cancer and Allied Diseases, Fred & Pamela Buffett Cancer Center, University of Nebraska Medical Center, Omaha, NE USA

**Keywords:** Targeted therapies, Cell growth

## Abstract

Acinar-to-ductal metaplasia (ADM) is a precursor lesion of pancreatic ductal adenocarcinoma (PDAC); however, the regulators of the ADM-mediated PDAC development and its targeting are poorly understood. RNA polymerase II-associated factor 1 (PAF1) maintains cancer stem cells leading to the aggressiveness of PDAC. In this study, we investigated whether PAF1 is required for the YAP1-mediated PDAC development and whether CA3 and verteporfin, small molecule inhibitors of YAP1/TEAD transcriptional activity, diminish pancreatic cancer (PC) cell growth by targeting the PAF1/YAP1 axis. Here, we demonstrated that PAF1 co-expresses and interacts with YAP1 specifically in metaplastic ducts of mouse cerulein- or Kras^G12D^-induced ADM and human PDAC but not in the normal pancreas. PAF1 knockdown (KD) reduced SOX9 in PC cells, and the PC cells showed elevated PAF1/YAP1 complex recruitment to the promoter of SOX9. The PAF1 KD reduced the 8xTEAD and SOX9 promoter-luciferase reporter activities in the mouse KC (Kras^G12D^; Pdx-1 Cre) cells and human PC cells, indicating that the PAF1 is required for the YAP1-mediated development of ADM and PC. Moreover, treatment with CA3 or verteporfin reduced the expressions of PAF1, YAP1, TEAD4, and SOX9 and decreased colony formation and stemness in KC and PC cells. CA3 treatment also reduced the viability and proliferation of PC cells and diminished the duct-like structures in KC acinar explants. CA3 or verteporfin treatment decreased the recruitment of the PAF1/YAP1 complex to the SOX9 promoter in PC cells and reduced the 8xTEAD and SOX9 promoter-luciferase reporter activities in KC and PC cells. Overall, PAF1 cooperates with YAP1 during ADM and PC development, and verteporfin and CA3 inhibit ADM and PC cell growth by targeting the PAF1/YAP1/SOX9 axis in vitro and ex vivo models. This study identified a regulatory axis of PDAC initiation and its targeting, paving the way for developing targeted therapeutic strategies for pancreatic cancer patients.

## Introduction

Pancreatic cancer (PC) is one of the most lethal diseases, and most PC cases are often diagnosed at later, more difficult-to-treat metastatic stages. Identifying the novel regulators of PC initiation and the therapeutic drug against these regulators is crucial to the early prognosis and intervention. The major pancreatic pre-precursor lesions derived from pancreatic acinar cells in response to the Kras^G12D^ mutation are acinar-to-ductal metaplasia (ADM) and pancreatic intraepithelial neoplasia (PanIN) [1].

Pancreatic differentiation 2 (PD2), also called PAF1 (RNA Polymerase II Associated Factor 1), is elevated in PC and regulates pancreatic cancer stem cells (PCSCs). PAF1 is one of the PAF1 complex (PAF1C) subunits, comprising other subunits (LEO1, CTR9, CDC73, SKI8). Studies have shown that the PAF1C regulates RNA polymerase II during transcriptional elongation in normal cells [2]. In contrast, studies from our lab demonstrated that the PAF1 acts independently of the complex, thereby regulating stemness features in embryonic stem cells and PCSCs [3–5]. Interestingly, the PAF1 expression was reduced during the ADM process; however, acino-ductal cells (intermediate cells in the ADM process) still retained and showed PAF1 expression [6]. Despite these studies, the mechanistic role of PAF1 in ADM and PC development remains unstudied.

Yes-associated protein 1 (YAP1) is elevated in PC and associated with PC cell proliferation and metastasis [7]. Oncogenic Kras promotes ADM by activating JAK-STAT3 signaling and ductal phenotype genes via YAP1-mediated activation [8]. A previous study has shown that the PAF1C component, CDC73 (Parafibromin) interacts with YAP1 leading to YAP1-mediated transcriptional activity in mouse fibroblasts and human embryonic kidney cells [9]. A specific subunit of PAF1C forms a sub-complex with other transcription factors, thereby exerting a particular function [3, 4]. However, the role of PAF1 in the YAP1-mediated ADM formation and PC development is unknown.

This study demonstrated that PAF1 is required for the YAP1-mediated PC development, and CA3 and verteporfin, inhibitors of YAP1/TEAD4 transcriptional activity, target the PAF1/YAP1/SOX9 axis to attenuate the development of PC.

## Methods

### Cell culture and treatments

The primary mouse cell line, KC6141, was generated from the pancreas of genetically engineered mice Kras^G12D^; Pdx-1 Cre (KC) [10]. The KC6141 cell line was cultured in RPMI-1640 (Invitrogen Cat# 11875) supplemented with 10% fetal bovine serum (FBS; R&D Systems Cat#S11550H) and 1% penicillin-streptomycin (Invitrogen Cat#15140-122). The SUIT2 cell line was a kind gift from Dr. Michel Ouellette, UNMC. MiaPaCa2 (CRL-1420) and human pancreatic ductal epithelial cell line (HPDE) were obtained from American Type Culture Collection (ATCC). The SUIT2 and MiaPaCa2 cell lines were cultured in Dulbecco’s Modified Eagle Medium (DMEM) media (Cytiva Cat#SH30022.01) supplemented with 10% FBS and 1% penicillin-streptomycin solution (Sigma-Aldrich). The HPDE cell line was cultured in human keratinocyte serum-free media supplemented with epidermal growth factor and bovine pituitary extract (Thermo Fischer Scientific, Waltham, MA). An immortalized human pancreatic nestin-positive epithelial (hTERT-HPNE) cell line was obtained as a generous gift from Dr. Ouellette at UNMC. HPNE cells were cultured in DMEM (low glucose) with 25% M3 Base Media (Incell), supplemented with 5% (v/v) FBS, 10 ng/ml EGF, and antibiotics (100 units/ml penicillin and 100 μg/ml streptomycin). All cell lines were authenticated and verified as mycoplasma-free every month. In some experiments, cells were treated with vehicle control (DMSO) or CA3 (Selleckchem Cat#S8661) (1 µM; 48–72 h) or verteporfin (MedChemExpress Cat#HY-B0146) (2 µM, 48–72 h).

### Mouse treatment studies

Animal experiments were carried out according to the UNMC Institutional Animal Care and Use Committee (IACUC) regulations. Mice were treated with cerulein (Sigma Cat# C9026) or PBS to induce acute pancreatitis as performed previously [6, 11].

### Immunofluorescence staining

Harvested tissues were fixed in 10% formalin and embedded in paraffin. Immunofluorescence analysis of tissue sections and cells seeded on coverslips was performed as shown previously [11, 12]. Whole-mount immunofluorescence staining for 3D collagen cultures was performed, as shown previously [13]. The primary antibodies used in this study include PAF1 1:100 (Invitrogen Cat# PA5-115713), mouse PAF1 1:100 (In house developed) [3], YAP1 1:100 (Santa Cruz Cat# sc-271134), TEAD4 1:100 (Invitrogen Cat#PA5-41446), TEAD1 1:100 (Invitrogen Cat#PA5-106477), SOX9 1:200 (Sigma-Aldrich Cat# AMAB90795) and CK19 1:50 (DSHB Hybridoma Product TROMA-III). In some immunofluorescence experiments, conjugated lectins, PNA-Rhodamine 1:100 (Vector Cat# RL-1072), and DBA-FITC 1:100 (Vector Cat# FL-1031) were used to stain acinar and ductal cells. Fluorescent images were captured using an LSM 710 confocal microscope and analyzed using ZEN software.

### Quantitative PCR

Total RNA was isolated using the RNeasy mini kit (Qiagen 74106). Reverse transcription was performed from 2 µg of total RNA utilizing an iScript cDNA synthesis kit (BioRad 1708890). Quantitative PCR was performed using SYBR Green dye (Roche 04887352001) using a CFX Connect Real-Time PCR detection system (BioRad 1855200). Reactions were performed in triplicate, and β-actin was used as a control. Human and mouse PAF1 and SOX9 primer sequences were obtained from our previous studies [3, 11]. The following primer pairs were used: CCN2 forward 5′-CATTCTCCAGCCATCAAG-3′ and CCN2 reverse 5′-CAAGCTGTCCAGTCTAATC-3′; AREG forward 5′-GTCCAGCTTAGAAGACAATAC-3′ and AREG reverse 5′-AGGACCGACTCATCATTT-3′; YAP1 forward 5′-CTGACCTGAAGGAGACCTAAGA-3′ and YAP1 reverse 5′-TGCTACCCAATACAACCAAGAA-3′; TEAD4 forward 5′-GACGAGGGCAAGATGTATGG-3′ and TEAD4 reverse 5′-CTTAGCTGCCTGGTCCTTTAG-3′.

Additional materials and methods for sphere culture, isolation of mouse primary pancreatic cells and 3D culture, immunoprecipitation, western blotting analysis, (ChiP)-re-ChiP assays, flow Cytometry, immunohistochemistry, cell proliferation, and colony formation assays, transfections and dual-luciferase reporter assay, IC50 calculations, and statistical analysis, are provided in the Supplementary Materials and Methods.

### Statistical analysis

Statistical analysis was performed using GraphPad Prism 8. Data are presented as the mean ± SD or mean ± SEM. Significance was determined using a simple student *t* test or one-way analysis of variance. A *p* value < 0.05 (*p* ≤ 0.05) was considered statistically significant.

## Results

### Expression correlation of PAF1 and YAP1 in normal human pancreas and PDAC progression tissue samples

**Fig. 1:**
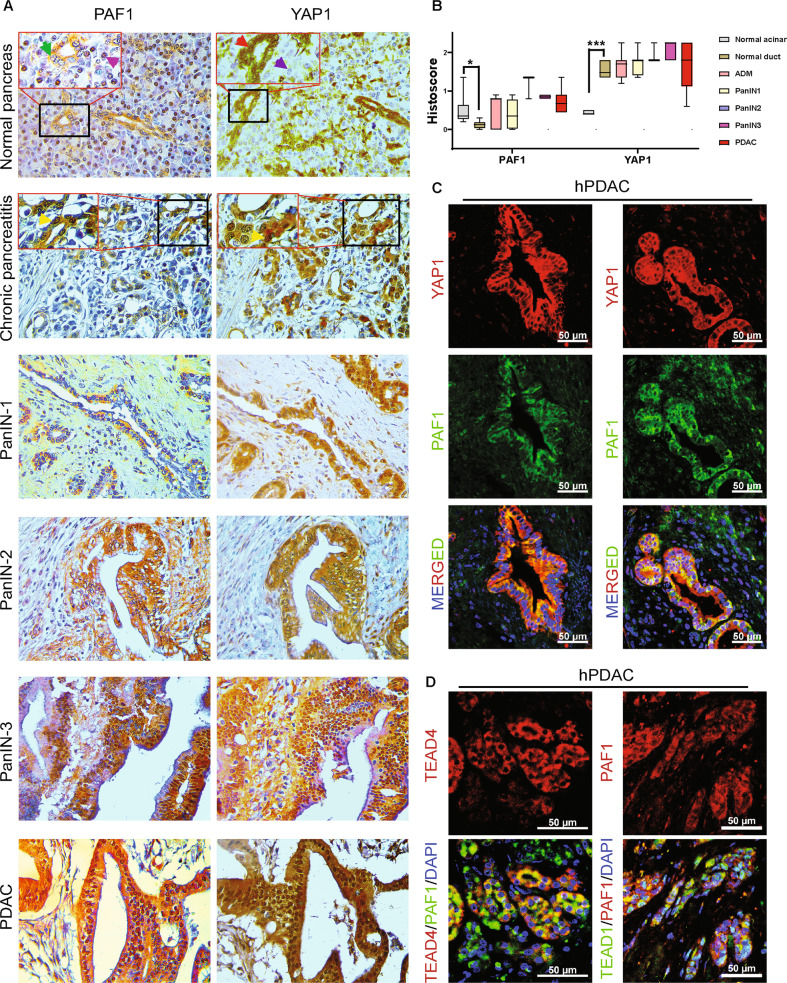
PAF1 co-expressed with YAP1 in human PanINs and PDAC samples but not in normal pancreas. **A** Immunohistochemical analysis of PAF1 and YAP1 in PDAC progression tissue samples. The green arrowhead in the magnified region of the normal pancreas points to the duct cell with no or low PAF1 staining, while the pink arrowhead in the magnified region points to the acinar cell with PAF1 staining. On the other hand, the red color arrowhead in the magnified region of the normal pancreas points to the duct cell with YAP1 staining, while the blue color arrowhead points to the acinar cell with no YAP1 staining. In addition, the yellow color arrowhead in chronic pancreatitis tissue samples points to the metaplastic ducts with PAF1 and YAP1 co-expression. Images were captured at ×20 magnification. **B** A histoscore was calculated by multiplying intensity and positivity. Data represent mean ± SD. p-values were calculated using ordinary one-way ANOVA (multiple comparisons). ^∗^*p* < 0.05, ^∗∗^*p* < 0.01, ^∗∗∗^*p* < 0.001. **C**, **D** Immunofluorescence images of human PDAC tissues stained with PAF1, YAP1, TEAD4, TEAD1, and DAPI. Scale bar 50 µm.

First, we performed a cBioportal analysis of the correlation of PAF1 with YAP1 in the pancreatic adenocarcinoma (QCMG, Nature 2016) dataset. Our studies revealed that the PAF1 expression is positively correlated with YAP1 (Supplementary Fig. 1). The immunohistochemical staining revealed that the normal pancreatic acinar cells show PAF1 expression; however, normal pancreatic duct cells show no expression of PAF1 (Fig. 1A). In contrast, YAP1 expression was observed in normal ducts but not in normal acinar cells (Fig. 1A). Interestingly, metaplastic ducts in chronic pancreatitis, PanINs, and pancreatic ductal adenocarcinoma (PDAC) showed a clear co-expression of PAF1 and YAP1. PAF1 expression was gradually elevated from chronic pancreatitis to PDAC (Fig. 1A, B). However, YAP1 staining intensity was not elevated from chronic pancreatitis/PanINs to PDAC. But the number of metaplastic ducts with YAP1 expression was elevated during the development of PDAC (from chronic pancreatitis/PanINs to PDAC). Immunofluorescent staining in human PDAC tissue sections showed a clear co-localization and co-expression of PAF1 with YAP1, TEAD4, and TEAD1 (Fig. 1C, D; Supplementary Fig. 2A–C). These observations suggest a strong link between PAF1 and YAP1/TEAD4 during the development and progression of PDAC.

### Co-expression of PAF1 and YAP1 in the ADM ducts of cerulein-induced acute pancreatitis mouse models

**Fig. 2:**
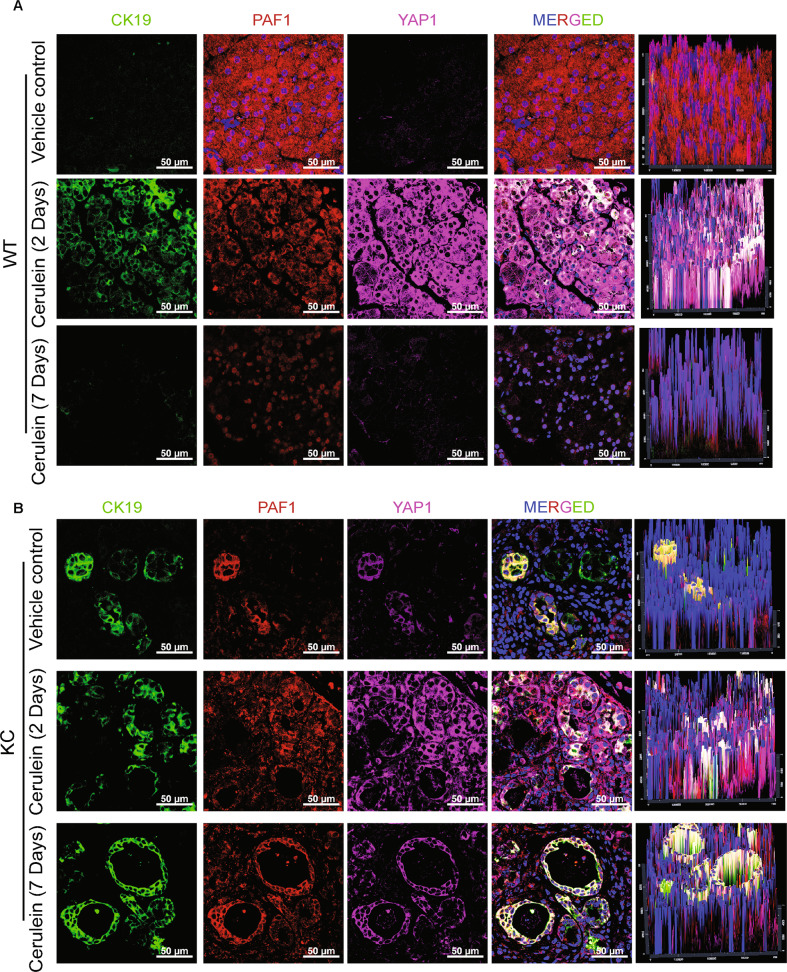
Co-expression of PAF1 with YAP1 in the pancreas tissues of cerulein-induced wild-type (WT) and Kras^G12D^; Pdx-1 Cre (KC) acute pancreatitis mouse models. **A**, **B** Immunofluorescence images of pancreas harvested from cerulein-treated WT and KC mice stained with PAF1, YAP1, and CK19. Pancreas tissues were harvested from WT and KC mice after 2 and 7 days of the last cerulein injection. Scale bar 50 µm.

We next focused on examining the expressional patterns of PAF1, YAP1, and TEAD1 in cerulein-induced wild-type (WT) and Kras^G12D^, Pdx-1 Cre (KC) pancreatitis mouse models [6, 11]. Similar to human PDAC samples (Fig. 1), the cerulein-induced WT and KC pancreas tissues (collected 2 days after the last cerulein injection) showed a clear co-expression of PAF1 with YAP1 and TEAD1 in metaplastic ducts (Fig. 2A, B; Supplementary Figs. 3 and 4). Interestingly, the cerulein-treated WT mouse pancreas collected 7 days after the last cerulein injection showed a recovery to the normal pancreas by losing YAP1 and TEAD1 expressions (Fig. 2A). This could probably be due to pancreas regeneration in normal WT pancreas. However, the pancreas of KC mice collected 7 days after the last cerulein injection showed persistent metaplastic ducts with the co-expression of PAF1 with YAP1 and TEAD1 (Fig. 2B), suggesting that the Kras^G12D^ mutation exacerbates the formation of ADM ducts with PAF1 and YAP1/TEAD co-expression.

### PAF1 interacts with YAP1 and regulates ductal phenotype through SOX9 in metaplastic ducts of KC mice pancreas and PC cell lines

**Fig. 3:**
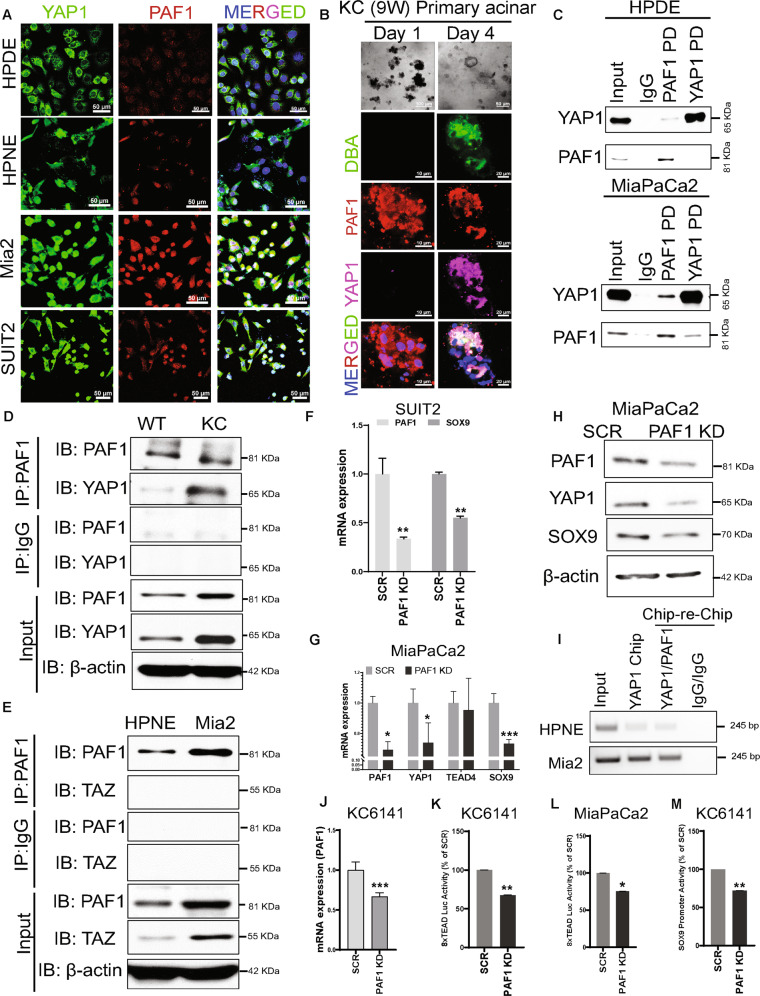
PAF1 interacts with YAP1 and regulates YAP1/TEAD4-mediated transcription and SOX9. **A** Immunofluorescence images. HPNE, HPDE, MiaPaCa2 (Mia2), and SUIT2 cells were stained with PAF1, YAP1, and DAPI. Scale bar, 50 µm. **B** Light microscope images: Isolated pancreatic acini from KrasG12D; Pdx-1 Cre (KC) mice were cultured in collage under 3D conditions (acinar explants embedded in collagen). Images were captured on days 1 and 4. Immunofluorescence images: Collagen discs with acinar explants were stained using immunofluorescence for PAF1, YAP1, and DBA (ductal-specific lectin). **C**–**E** Immunoprecipitation assay. PAF1 or YAP1 pulldown followed by immunoblotting with YAP1 or PAF1 or TAZ in indicated samples. **F**, **G** Quantitative real-time PCR (qRT-PCR) analysis of PAF1, SOX9, YAP1, and TEAD4 in PAF1 KD and scramble control PC cells. qRT-PCR data were normalized with the Actb gene. **H** Western blot analysis of PAF1, YAP1, and SOX9 expression in PAF1 KD and scramble control MiaPaCa2 cells. β-actin was used as a control. **I** ChiP-re-ChiP assay was performed using PAF1, YAP1, and control IgG antibodies in HPNE and MiaPaCa2 (Mia2) cells using primers that amplify SOX9 promoter, which contains TEAD binding motif. Immunoprecipitated and purified DNA was amplified using PCR followed by running in agarose gel. **J** qRT-PCR analysis of PAF1 in PAF1 KD and scramble control KC6141 cells. qRT-PCR data were normalized with the Actb gene. **K**–**M** 8xTEAD luciferase, and SOX9 promoter activity luciferase assays in indicated cells. The bar graphs show activities of 8xTEAD synthetic, and SOX9 promoters represented as the percentage of control (scramble). For all graphs, data are mean ± SD, *n* = 3. For all panels, significance was determined with a student’s *t* test. *p < 0.05, **p < 0.01, ***p < 0.001, ns: non-significant, *p* > 0.05.

Immunofluorescence staining showed that the normal human pancreatic duct epithelial (HPDE) cells and HPNE cells exhibit high YAP1 expression but low PAF1 expression; however, PC cells (SUIT2 and MiaPaCa2) show elevated YAP1 and PAF1 expression and co-localization (Fig. 3A and Supplementary Fig. 5). The primary acinar cells isolated from KC mice pancreas grown as explants embedded in collagen showed duct-like structures after 4 days (Fig. 3B; Supplementary Fig. 6). Immunofluorescent staining on these collagen discs revealed that the primary acinar cells on day 1 express PAF1 without YAP1 expression; however, after 4 days, the duct-like structures show a clear co-expression of PAF1 and YAP1 (Fig. 3B). The immunoprecipitation of PAF1 or YAP1 followed by immunoblotting with YAP1 or PAF1 showed a clear interaction between these two molecules in PC (MiaPaCa2) cells; however, this interaction was not observed in HPDE normal cells (Fig. 3C). Similarly, PAF1 interacted with YAP1 only in the pancreas isolated from KC mice (9 weeks old), but not in the pancreas of WT mice (Fig. 3D). Interestingly, PAF1 did not show interaction with TAZ in PC cells (Fig. 3E). These findings suggest that PAF1 interacts with YAP1 during the formation of ADM and the development of PC. In addition, PAF1 knockdown (KD) decreased the expression of YAP1, AREG (YAP1 target gene), and SOX9 (Fig. 3F–H; Supplementary Fig. 7). YAP/TEAD complex binds to the TEAD binding motif (^−281^CATTCC^−275^) on the proximal promoter of SOX9 gene and transcriptionally regulates its expression [14]. Hence, we next sought to examine whether the PAF1/YAP1 complex is recruited to the SOX9 gene promoter. The chromatin immunoprecipitation (ChIP)-re-ChIP assay showed that the recruitment of PAF1/YAP1 complex to SOX9 promoter is more in PC cells compared to normal pancreatic cells (Fig. 3I). The reporter activities of 8xTEAD and SOX9 promoter were also reduced with PAF1 KD in MiaPaCa2 and KC6141 cells (Fig. 3J–M). Overall, these results indicate that PAF1 is required for YAP1/TEAD-mediated transcription and SOX9 transcriptional activation during PC development.

### Treatment with inhibitors of YAP/TEAD transcriptional activity reduces PAF1 and decreases cancer stem cells, cell viability, proliferation, and colony formation in PC

**Fig. 4:**
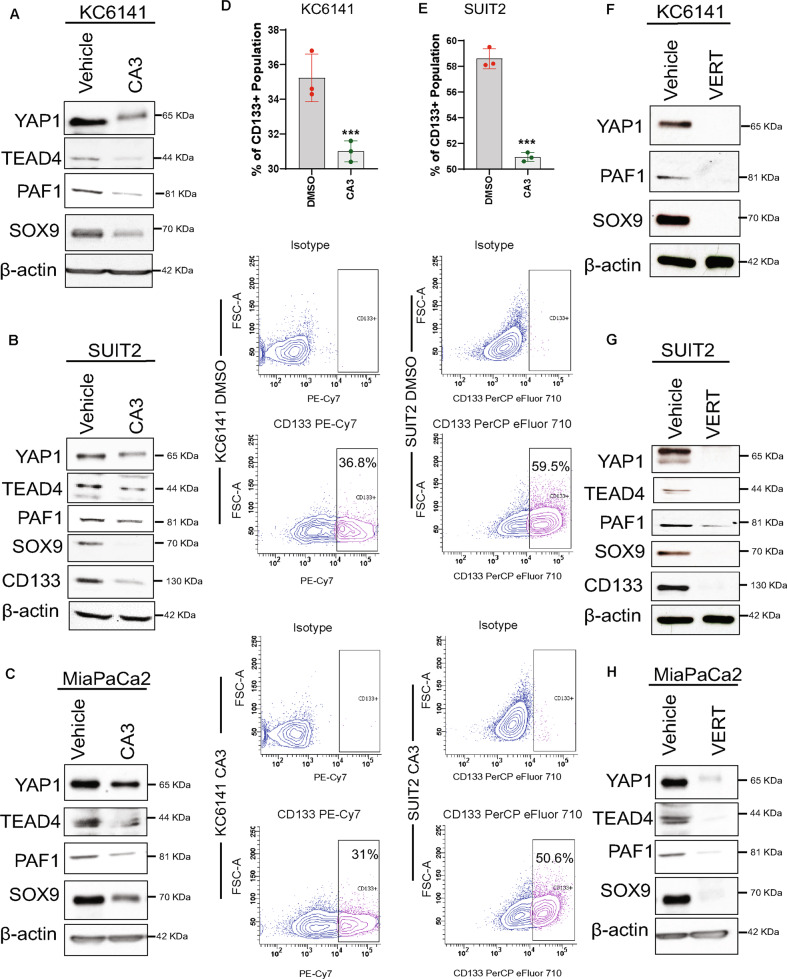
CA3 and verteporfin inhibit YAP1/TEAD4/PAF1 and reduce cancer stem cell markers and features. **A**–**C**, Western blot analysis of protein expression levels of PAF1, YAP1, TEAD4, SOX9, and CD133 in indicated cell lines treated with vehicle control or CA3 (1 µM, 48 h). β-actin was used as a control. **D**, **E** Flow cytometry analysis of CD133+ population in KC6141 and SUIT2 cell lines with or without CA3 treatment. Bar graphs show the percentage of the CD133+ population. Data are mean ± SD, *n* = 3. **F**–**H** Western blot analysis of protein expression levels of PAF1, YAP1, TEAD4, SOX9, and CD133 in indicated cell lines treated with vehicle control or verteporfin (2 µM, 48 h). β-actin was used as a control. For all panels, significance was determined with a student’s *t* test. *p < 0.05, **p < 0.01, ***p < 0.001, ns non-significant, *p* > 0.05.

CA3, an inhibitor of YAP/TEAD transcriptional activity, inhibits the proliferation and metastasis of other cancers [15, 16]; however, whether it inhibits PAF1-mediated YAP1/TEAD4 transcriptional activity and cancer stem cells in PC is unknown. Investigation of IC50 values revealed that the 9–26 NP (normal human fibroblast cell line) has a higher IC50 value (1612 nM) than the SUIT2 PC cell line (1097 nM) and MiaPaCa2 PC cell line (913.49 nM) (Supplementary Figs. 8A, B and 9). CA3 treatment (1 µM; 48 h) in PC cells reduced the protein expression levels of PAF1, YAP1, TEAD4, CD133 (cancer stem cell marker), and SOX9 (pancreatic ductal marker) (Fig. 4A–C). Moreover, the CA3 treatment inhibited the CD133+ PC stem cell population and side population (drug-resistant cells) (Fig. 4D, E; Supplementary Fig. 10). Next, we sought to see whether verteporfin, another inhibitor of YAP1, also has the potential to inhibit PAF1-mediated YAP/TEAD transcriptional activity. The IC50 value of verteporfin for the MiaPaCa2 cell line was 2182.47 nM (Supplementary Fig. 11). Interestingly, verteporfin treatment (2 µM; 48 h) also inhibited the protein expression levels of PAF1, YAP1, TEAD4, CD133, and SOX9 in KC and PC cells (Fig. 4F–H).

**Fig. 5:**
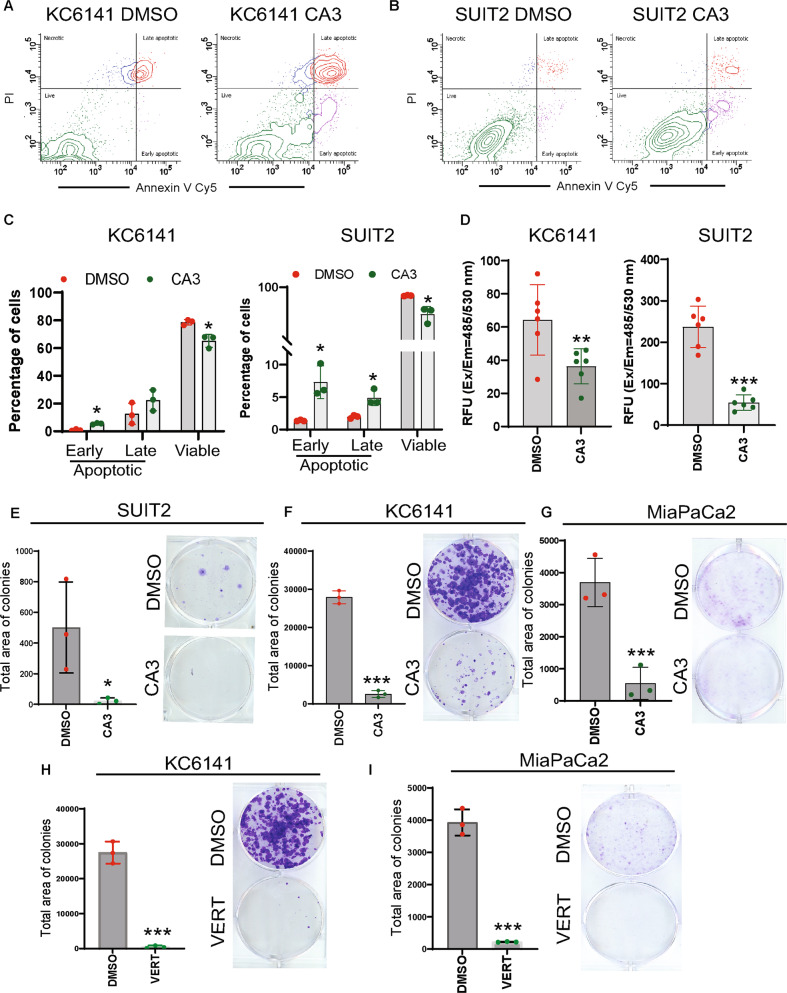
CA3 and verteporfin diminish PC promotion in vitro. **A**–**C** Flow cytometry analysis of apoptosis. Cells were stained with Annexin V Cy5 and propidium iodide (PI), followed by flow cytometry analysis. Bar graphs show the percentage of apoptotic and viable cells in vehicle control and CA3-treated KC6141 and SUIT2 cells. Data are mean ± SD, *n* = 3. **D** Calcein-AM-based proliferation assay. Bar graphs show the relative fluorescence units (RFU). Data are mean ± SD, *n* = 6. **E**–**I** Colony formation assay. Five hundred cells (SUIT2, MiaPaCa2, and KC6141) were seeded in six-well plates and allowed to form colonies. These colonies were exposed to vehicle control or CA3 (1 µM, 72 h) or verteporfin (2 µM, 72 h). Bar graphs show the total area of the colonies. Data are mean ± SD, *n* = 3. For all panels, significance was determined with a student’s *t* test. *p < 0.05, **p < 0.01, ***p < 0.001, ns: non-significant, *p* > 0.05.

Treatment with CA3 increased the percentage of early and late apoptotic cells and reduced the percentage of viable PC cells (Fig. 5A–C). The PC cells treated with CA3 also showed reduced proliferation (Fig. 5D), colony formation (Fig. 5E–G), and tumorsphere formation (Supplementary Fig. 12). Interestingly, treatment with verteporfin (2 µM; 72 h) also drastically reduced the colony formation in PC cells (Fig. 5H, I). Overall, CA3 and verteporfin have the potential to inhibit cancer stemness and cell growth in PC.

### **CA3 and verteporfin target the PAF1/YAP1/SOX9 axis and inhibit PC development**

**Fig. 6:**
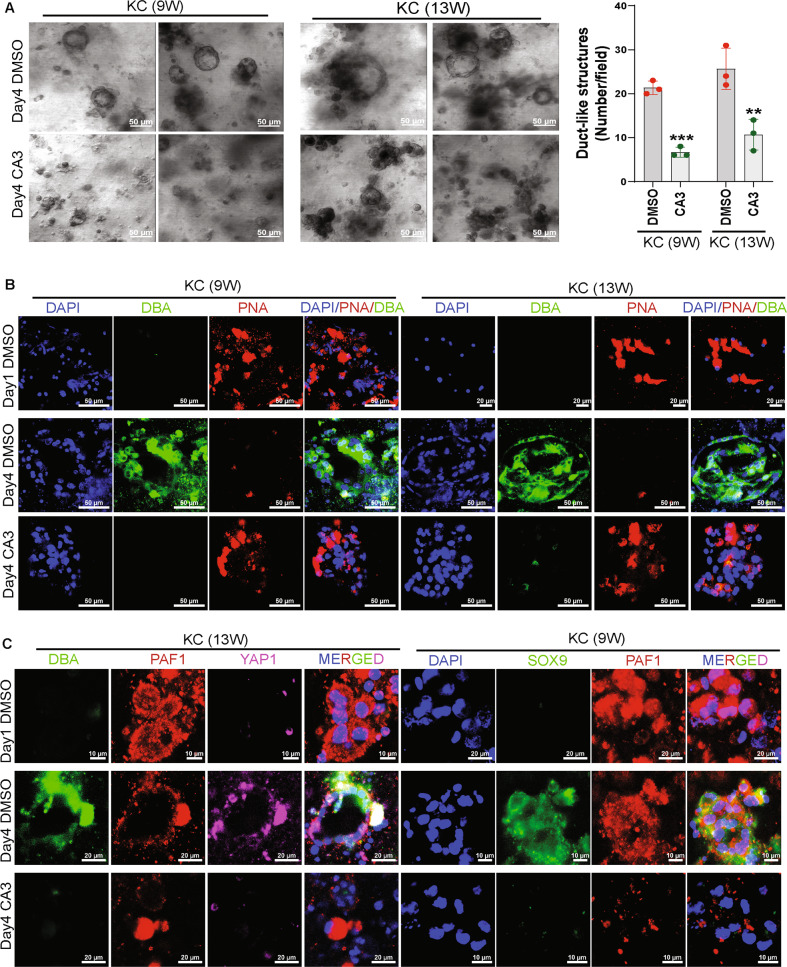
CA3 inhibits ADM, SOX9 expression, and PAF1/YAP1 co-expression. **A** Light microscope images of pancreatic acinar cell explants from KC mice embedded in collagen treated with vehicle control or CA3 for 72 hr. The bar graph shows the number of duct-like structures per field. Data are mean ± SD, *n* = 3. Significance was determined with a student’s *t* test. *p < 0.05, **p < 0.01, ***p < 0.001, ns: non-significant, *p* > 0.05. **B**, **C** Confocal images of whole-mount immunofluorescence staining on the acinar explants embedded in collagen treated with vehicle control or CA3. Explants in collagen discs were stained with FITC-labeled Dolichos biflorus agglutinin (DBA), a duct-specific lectin, and rhodamine-labeled Peanut agglutinin (PNA), an acinar-specific lectin, and antibodies for PAF1, YAP1, and SOX9.

**Fig. 7:**
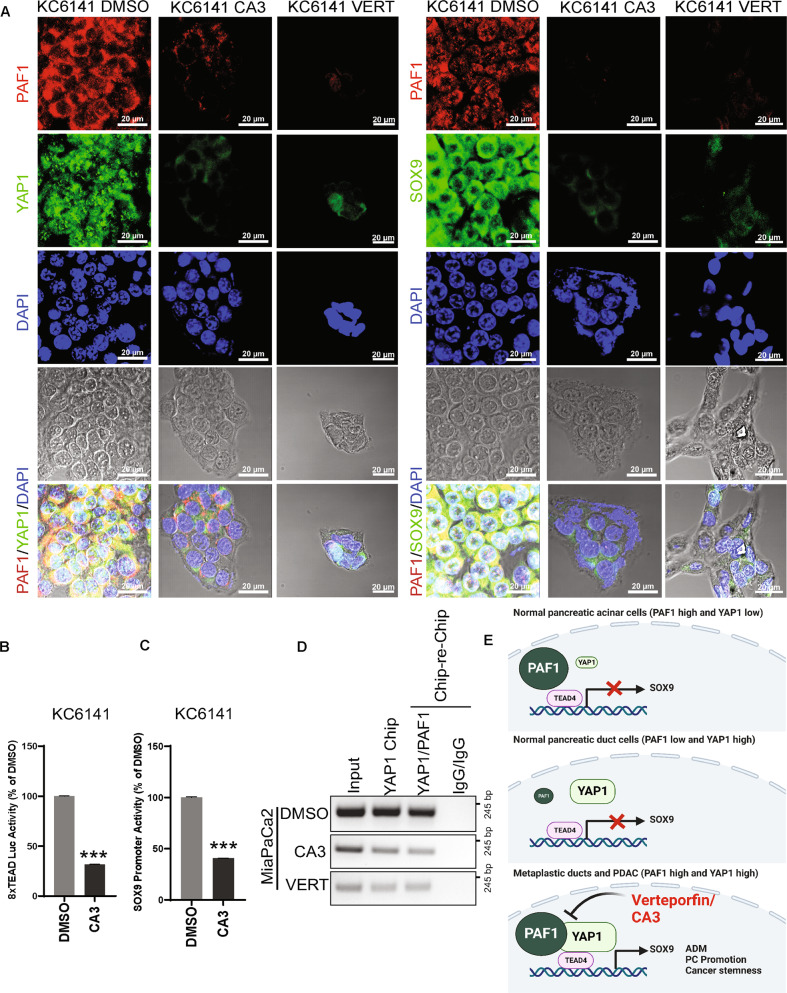
CA3 and verteporfin inhibit the PAF1/YAP1/TEAD4-mediated transcriptional activation of SOX9 in PC development. **A** Confocal images of immunofluorescence staining for PAF1 co-expression with YAP1 and SOX9 in the presence and absence of CA3 (1 µM, 48 h) or verteporfin (2 µM, 48 h) treatment in KC6141 cells. **B**, **C** 8xTEAD luciferase and SOX9 promoter activity luciferase assays KC cells treated with CA3 or vehicle control. The bar graphs show activities of 8xTEAD synthetic and SOX9 promoters represented as the percentage of control (DMSO). Data are mean ± SD, *n* = 3. Significance was determined with a student’s *t* test. *p < 0.05, **p < 0.01, ***p < 0.001, ns: non-significant, *p* > 0.05. **D** ChIP-re-ChIP assay was performed in MiaPaCa2 cells treated with DMSO or CA3 (1 µM, 48 h) or verteporfin (2 µM, 48 h) using PAF1, YAP1, and control IgG antibodies using primers that amplify SOX9 promoter. Immunoprecipitated and purified DNA was amplified using PCR followed by agarose gel electrophoresis. **E** Schematic shows the overall summary of the study.

The pancreatic KC acinar explants embedded in collagen were treated with CA3 and observed that CA3 significantly reduces the duct-like structure (Fig. 6A). Whole-mount immunofluorescence staining on the CA3- or vehicle control-treated explant-collagen discs showed that the CA3 treatment maintains the acinar phenotype (PNA-Rhodamine staining) and inhibits the ductal phenotype (DBA-FITC staining) (Fig. 6B). CA3 also reduced the immunofluorescence staining for PAF1 and YAP1 in day 4 acinar explants embedded in collagen (Fig. 6C). Immunofluorescence staining on primary KC cells treated with CA3 or verteporfin showed that these inhibitors reduce the staining for PAF1, YAP1, TEAD4, and SOX9 (Fig. 7A; and Supplementary Fig. 13). Interestingly, 8xTEAD luciferase activity and SOX9 luciferase reporter activities were reduced with CA3 treatment in KC cells (Fig. 7B, C). The ChIP-re-ChIP assay further showed that the CA3 and verteporfin impeded the recruitment of PAF1/YAP1 to the SOX9 promoter (Fig. 7D). Our data indicate that CA3 and verteporfin inhibit the PAF1, YAP1, and TEAD4 leading to the transcriptional inactivation of the SOX9 gene (Fig. 7E).

## Discussion

One of the major features of PDAC is its early advancement to metastasis; therefore, this disease is often diagnosed with advanced metastatic stage and has a poor survival rate. Identifying the molecular mechanisms, novel regulators of PC initiation, and the drugs that target these regulators is crucial to improving prognosis and patient survival.

We have previously demonstrated that PAF1 functions independently of its complex molecules by forming a sub-complex with other transcription factors [3]. Tang et al. have shown that the PAF1 complex component, CDC73, interacts with YAP1 resulting in the activation of canonical YAP1-mediated transcriptional activity in mouse fibroblasts and human embryonic kidney cells [9]. However, whether PAF1 cooperates with YAP1 through their interaction to induce ADM and PC development is unkown. Our data indicated that the pancreatic acinar and ductal cells show differential expression of PAF1 in the normal pancreas. PAF1 expression was localized to normal acinar cells but not to normal duct cells. In contrast, YAP1 expression was localized to the ductal component of the normal pancreas but not to the acinar cells. Similarly, Morvaridi et al. have also shown the ductal localization of YAP1 in the normal pancreas [17]. Interestingly, PAF1 and YAP1 were co-localized and co-expressed in metaplastic ducts and PDAC tumor tissues. Similarly, human pancreatic normal ductal epithelial cells showed YAP1 expression with low PAF1 expression; however, PC cells showed an elevated co-overexpression of PAF1 and YAP1. PAF1 was also co-expressed with TEAD4 and TEAD1 in PDAC tissues. These data indicate the potential of PAF1 and YAP1 cooperation in PC initiation and PDAC. Our data also showed that PAF1, YAP1, and TEAD are co-expressed in cerulein-induced metaplastic ducts and ADM in wild-type and KC mice pancreas collected 2 days after the last cerulein injection. The co-expression of PAF1 with YAP1 and TEAD1 was lost in wild-type mice pancreas 7 days after the last cerulein injection, owing to acinar regeneration [18]; however, the pancreas of cerulein-induced KC mice collected after 7 days of recovery showed a persistent ADM and metaplastic ducts with PAF1 co-expression with YAP1 and TEAD1. Studies showed that Kras^G12D^ induces the formation of duct-like structures spontaneously from acinar explants embedded in collagen [13, 19]. The pancreatic acini explants embedded in collagen for 4 days showed a clear co-expression of PAF1 and YAP1, while these acini on day 1 did not show this co-expression. These observations indicate that the mutant Kras induces persistent ADM with PAF1 and YAP1/TEAD expression.

Next, we identified that PAF1 interacts with YAP1 in the KC and PC cells but not in the WT and normal human pancreatic cells. Notably, PAF1 did not exhibit any interaction with TAZ. A previous study demonstrated that the activation of YAP and TAZ during PC development results in the transcriptional activation of STAT3 [8]. It was also shown that the YAP1 and TAZ have specific binding motifs on the target genes with specific biological functions [20]. Our data suggest that PAF1 KD decreases the expression of SOX9 and AREG (YAP1 target gene) in PC cells. However, PAF1 KD did not affect the expression of CCN2, the other YAP1 target gene. Moreover, tyrosine phosphorylated Parafibromin, a PAF1 complex protein, interacts with YAP1 and activates YAP1-mediated transcription. In contrast, tyrosine dephosphorylated Parafibromin interacts with TAZ/β-catenin, activating TAZ/β-catenin-mediated target gene transcription [9] in mouse fibroblasts and human embryonic kidney cells. Taken together, these studies and our observations suggest that YAP1 and TAZ may act independently as well as in conjunction and that YAP and TAZ, either in conjunction or separately, interact with other factors to regulate specific target genes: a concept that needs to be further studied in the future. Thus, future research must also determine whether PAF1/YAP1/TEAD-mediated SOX9 gene transcription in PC is independent of TAZ. But our data concludes that PAF1 interacts with YAP1 and regulates SOX9. It was shown that the YAP1/TEAD complex transcriptionally regulates the SOX9 gene in esophageal cancer [14]. Hence, we hypothesized that PAF1 cooperates with YAP1/TEAD at the transcriptional level to control the SOX9 gene. Our data showed the binding of the PAF1/YAP1 complex to the TEAD binding motif on the proximal promoter region of the SOX9 gene. Moreover, PAF1 KD reduced the TEAD luciferase reporter and SOX9 promoter reporter activities in KC and PC cells. Interestingly, PAF1 silencing in PC cells also reduced the expression of YAP1, indicating that PAF1 is required for the stability of the complex. These data suggest that the PAF1 is necessary for the YAP1/TEAD-mediated transcriptional activation of SOX9.

Currently, no clinically promising drugs are available to target the YAP1-mediated development of cancers, including PC. In addition, the drugs that target early-stage of PDAC development are poorly developed. Previous studies have shown that CA3, a novel YAP1/TEAD4 transcriptional activity inhibitor, targets cancer stem cells in esophageal adenocarcinoma and mesothelioma [15, 16]. We observed reduced protein expression levels of YAP1, TEAD4, PAF1, SOX9, and CD133 with CA3 treatment in PC cells. Furthermore, CA3 induced apoptosis and reduced KC and PC cell proliferation and colony formation. The number of duct-like structures was significantly reduced with CA3 treatment in isolated acini explants embedded in collagen. CA3 treatment also reduced the PAF1 and YAP1 staining in acini from Kras^G12D^ mice cultured in collagen. KC cells also showed reduced staining for PAF1, YAP1, and TEAD4 with CA3 treatment. CA3 treatment reduced the binding of the PAF1/YAP1 complex to the TEAD binding motif on the SOX9 promoter and also reduced the TEAD luciferase and the SOX9 promoter-luciferase reporter activities. Another YAP1 inhibitor, verteporfin, targeted the YAP1/TEAD complex and inhibited cancer stemness and cell growth in esophageal cancer and PC [14, 21]. Notably, verteporfin treatment showed a drastic reduction in the protein expression levels of PAF1, YAP1, TEAD4, SOX9, and CD133 compared to CA3 in KC and PC cells. Compared to CA3, verteporfin treatment significantly decreased colony formation and diminished the recruitment of the PAF1/YAP1 complex to the TEAD binding site on the SOX9 promoter. Both CA3 and verteporfin target the PAF1/YAP1/SOX9 axis to alleviate PC development in vitro and in ex vivo models. However, verteporfin has the highest potential in targeting the PAF1/YAP1/SOX9 axis and PC cell growth. Verteporfin increases the expression of 14-3-3σ, a YAP chaperon protein, which increases the cytoplasmic retention of YAP1 and inhibits its transcriptional activity [22]. The mechanism by which verteporfin reduces PAF1 needs further research to develop a novel verteporfin-based therapeutic strategy for targeting PAF1. Overall, this study revealed the role of PAF1 in the YAP1-mediated ADM and PC development and showed that PAF1 cooperation with YAP1 is critical for PC development.

## Supplementary information

Supplementary Materials and Methods

Supplementary Fig1

Supplementary Fig2

Supplementary Fig3

Supplementary Fig4

Supplementary Fig5

Supplementary Fig6

Supplementary Fig7

Supplementary Fig8

Supplementary Fig9

Supplementary Fig10

Supplementary Fig11

Supplementary Fig12

Supplementary Fig13

Original Data File

Reproducibility checklist

## Data Availability

The data generated or analyzed during this study are available from the corresponding author upon reasonable request. No applicable resources were generated during the current study.
