# Supplementary material for: PAF1 cooperates with YAP1 in metaplastic ducts to promote pancreatic cancer
Source: Cell Death Dis. 2022 Oct 1;13(10):839. doi: 10.1038/s41419-022-05258-x (PMC9525575; doi:10.1038/s41419-022-05258-x)
Supplement: Supplementary file 1 — Supplementary Materials and Methods [file 41419_2022_5258_MOESM1_ESM.docx]

**PAF1 cooperates with YAP1 in metaplastic ducts to promote pancreatic cancer**

Rama Krishna Nimmakayala^1^, Ayoola O. Ogunleye^1^, Seema Parte^1^, Nivedeta Krishna Kumar^1^, Pratima Raut^1^, Venkatesh Varadharaj^1^, Naveen Kumar Perumal^1^, Palanisamy Nallasamy^1^, Sanchita Rauth^1^, Jesse L. Cox^2^, Subodh M. Lele^2^, Surinder K. Batra^1,3^, and Moorthy P. Ponnusamy^1,3^.

^1^Department of Biochemistry and Molecular Biology, College of Medicine, University of Nebraska Medical Center, Omaha, NE 68198-5870, USA

^2^Department of Pathology and Microbiology, College of Medicine, University of Nebraska Medical Center, Omaha, NE

^3^Eppley Institute for Research in Cancer and Allied Diseases, Fred & Pamela Buffett Cancer Center, University of Nebraska Medical Center, Omaha, NE

**Supplementary Materials and Methods**

**Supplementary References**

**Sphere culture**. For sphere culture, cells were cultured in 96-well low-attachment culture plates in stem cell medium: DMEM/F12 (Invitrogen Cat#11330-032) supplemented with 1% B27 (Invitrogen Cat# 17504-044), epidermal growth factor (20 ng/mL), and basic fibroblast growth factor (10 ng/mL). The number of spheres for each well was monitored for 4 to 10 days. Images were captured using the EVOS FL Auto Imaging System (Thermo Fisher Scientific). The number of spheres (>50 µm) was counted per field (whole well). In some experiments, spheres were treated with vehicle control (DMSO) or CA3 (1 µM) for two days before analysis.

**Immunoprecipitation:** Immunoprecipitation assay was performed using Dynabeads Protein G immunoprecipitation kit (Invitrogen Cat#10007D). Cells were lysed in IP lysis buffer (Thero Scientific Cat#87787) mixed with Halt protease and phosphatase inhibitor cocktail (Invitrogen Cat#78440). Protein lysates (500 µg) were immunoprecipitated with 2 µg of rabbit anti-PAF1 antibody (Abcam Cat#ab20662) or rabbit anti-YAP1 antibody (Invitrogen Cat# PA1-46189). First, 20 µl of dynabeads were incubated with rabbit anti-PAF1 antibody or rabbit anti-YAP1 antibody in ‘antibody binding and washing buffer’ for 1 h at room temperature. After washing, the antibody-bound beads were incubated with protein lysates overnight at 4°C. The next day, the immune complex on beads was washed three times with washing buffer, and the sample was eluted in elution buffer. The eluted samples were mixed with 5x Lane Marker Non-Reducing Sample Buffer (Thermo Scientific Cat#39001) followed by heating the samples at 95^0^C for 5 min before loading in 10% SDS-PAGE gel. In some experiments, elution was performed in elution Buffer pre-mixed with NuPAGE™ LDS Sample Buffer (Thermo Scientific Cat# NP0007) and NuPAGE Sample Reducing Agent (Thermo Scientific NP0009) followed by heating for 10 min at 70ºC before loading the sample into the gel. Thus, the immunoprecipitated products were subjected to 10% SDS-PAGE, then transferred onto a PVDF membrane and immunoblotted with rabbit anti-YAP1 antibody 1:1000 (Invitrogen Cat# PA1-46189) or rabbit anti-PAF1 antibody 1:5000 (Bethyl Laboratories Cat#A300-172A) or rabbit anti-TAZ antibody 1:1000 (Invitrogen Cat#703032). After washing, the membranes were incubated with Goat anti-rabbit IgG (H+L) secondary antibody, HRP (Invitrogen Cat#31460) or Clean-Blot IP detection kit (HRP) (Invitrogen Cat#21232).

**Western blotting analysis**. Cells were lysed in RIPA buffer (50 mM Tris-HCl, 150 mM NaCl, 1% NP-40, 0.5% sodium deoxycholate, 0.1% sodium dodecyl sulphate [SDS]) containing protease inhibitors (1 mM phenyl-methyl sulphonyl fluoride, 1 µg/ml aprotinin, 1 µg/ml leupeptin). Cell lysates were spun at 13,000 rpm for 30 min to remove debris, and protein quantification was performed using the Bio-Rad DC Protein Assay kit (RRID:SCR_008426). Total protein (40 μg/well) was fractionated by 10% SDS–PAGE. Fractionated proteins were transferred to polyvinylidene difluoride (PVDF) membranes. Membranes were blocked in 5% non-fat dry milk in PBS containing 0.1% Tween 20 (PBST). Blots were incubated overnight at 4°C with primary antibodies: YAP1 (1:1000, Proteintech Cat# 13584-1-AP), YAP1 (1:1000, Invitrogen Cat# PA1-46189), TEAD4 (1:1000, Invitrogen Cat# PA5-41446), PAF1 (1:5000, Bethyl Laboratories Cat#A300-173A), SOX9 (1:1000, Abcam Cat#ab185966), and CD133 (1:1000 Abcam Cat#ab19898). β-actin was used as a loading control for protein normalization. The membranes were then washed in PBST, probed with the appropriate secondary antibodies, incubated for an hour at room temperature, and then washed with PBST. Signals were detected with the Pierce ECL Western Blotting Substrate kit (Thermo Scientific).

**Chromatin immunoprecipitation (ChiP)-re-ChiP assay.**

ChiP assay was performed as shown previously ^1^ with few modifications. Cells were fixed with 0.4% formaldehyde and 1.5 mM EGS (ethylene glycol bis (succinimidyl succinate)) (Thermo Scientific Cat#21565), washed, collected, and resuspended in 500 μl SDS lysis buffer (1% SDS, 10 mM EDTA, 50 mM Tris‐HCl [pH 8.1], 1 mM PMSF, and 1 μg/ml aprotinin). Then, the samples were sonicated and diluted in ChIP dilution buffer (0.01% SDS, 1.1% Triton X‐100, 1.2 mM EDTA, 16.7 mM Tris‐HCl [pH 8.1], 167 mM NaCl, 1 mM PMSF, and 1 μg/ml aprotinin). 10% of sonicated samples were separated for the input control. Immunoprecipitation was performed with ChIP‐grade rabbit anti-PAF1 antibody (Abcam Cat#ab20662) or rabbit anti-YAP1 antibody (Invitrogen Cat# PA1-46189). Chromatin was immunoprecipitated in a sequential manner with rabbit anti-YAP1 antibody (Invitrogen Cat# PA1-46189) and rabbit anti-PAF1 antibody (Abcam Cat#ab20662). Chromatin extracts were pulled down with Dynabeads Protein G. The samples were washed extensively with wash buffers (low salt, high salt, LiCl, and Tris/EDTA buffers), eluted with SDS elution buffer, and subjected to reverse cross-linking and proteinase digestion. As a control, IgG antibody was used for the ChIP assay. The captured and purified DNA was subjected to PCR amplification using the SOX9 gene promoter primers spanning the TEAD binding site as shown in a previous study ^2^: SOX9 forward 5′-GTCCCCGGTGCCGCGGAGAGAGC-3’ and SOX9 reverse: 5’-GGGATCGCAGCCAAAGGGCGGAC-3’.

**Flow Cytometry.**

Analysis of stem cell surface markers: One million cells in 100 µL buffer (PBS supplemented with 2% fetal bovine serum) were stained with the following antibodies: CD133 PE Cy7 (0.5 µg/100 µl/1 million cells) (Biolegend Cat# 141210) or CD133 PerCP-eFluor710 (0.2 µg/100 µl/1 million cells) (Invitrogen Cat#46-1331-82). After two washes in PBS, cells were counterstained with DAPI to exclude dead cells.

Side population (SP) assay: SP analysis was performed with flow cytometry using Hoechst 33342 (AnaSpec Inc., Fremont, CA, USA) (5 µg/ml). Verapamil (Sigma) control, an inhibitor of ABC transporters, was used for SP identification at a 100 µM final concentration.

Apoptosis assay: Cells were treated with DMSO or CA3 (1µM) for 48 hours. Cell supernatant and adherent cells were collected, stained with annexin V and propidium iodide, and analyzed by flow cytometry.

**Immunohistochemistry**. Tissue microarray (TMA), CP (US Biomax Cat# BIC14011b) was purchased and the tissue samples from the normal pancreas (n=6), chronic pancreatitis (CP) (n=16), PanIN1 (n=8), PanIN2 (n=3), PanIN3 (n=6), and PDAC (n=8) are included in the TMA. The TMA was subjected to immunohistochemical staining by using the following primary antibodies at the indicated dilution: PAF1 1:100 (Invitrogen Cat#MA5-38068) and YAP1 1:100 (Proteintech Cat# 13584-1-AP). Antigen retrieval was performed in 10 mM sodium citrate buffer (pH 6). The stained sections were scored in a blinded fashion by Dr. Cox and Dr. Lele (pathologists at UNMC). The intensity of protein expression was graded on a scale of 0 to 3 (0, no staining; 1+, weakly positive; 2+, moderately positive; 3+, strongly positive). The percentage of positive staining was scored in a range of (0–100% or 0–1). A histoscore was calculated by multiplying intensity (0–3) and positivity (0–1), ranging between 0 and 3. GraphPad Prism software (RRID:SCR_002798) was used to calculate P values and to design graphs.

**Isolation of mouse primary pancreatic cells and 3D culture.** The pancreas was removed and washed with ice-cold HBSS media. The organ was minced into 1–5-mm pieces, followed by digestion with collagenase P (HBSS media containing 10 mM HEPES, 0.5 mg/mL of collagenase- P (Millipore Sigma Cat# COLLP-RO Roche), 10 µg/mL of DNase I, and 0.2 mg/ml of trypsin inhibitor (Thermofisher Scientific, Cat# 17075029). An equal volume of cold HBSS media containing 10% FBS was added after digestion to stop the digestion. The digested pancreatic pieces were washed twice with HBSS media containing 10% FBS and pipetted through a 100-µm cell strainer (Corning Cat# CLS431752). The filtered cell suspension was centrifuged through HBSS supplemented with 30% FBS at 1000 rpm for 2 min. The pellet was resuspended in RPMI supplemented with 100 mg/ml gentamicin, 2% FBS, 0.1 mg/ml soybean trypsin inhibitor (Sigma Cat#T6522), and 1 mg/ml dexamethasone. A 1:1 ratio of cell suspension and rat tail collagen type 1 (RTC, Gibco Cat# A1048301) was added to the pre-coated collagen layers in 24 well plates, followed by incubation at 37ºC, 5% CO_2_ for 30 min to allow the collagen to solidify. RPMI medium supplemented with appropriate growth factors or inhibitors was added.

**Cell proliferation and colony formation assays.** For proliferation assay, 10,000 cells were seeded in 96 well plates, followed by treatment with vehicle control and CA3 (1 µM) for 48 h. Cells were stained with Calcein AM (Invitrogen Cat#C3100MP) followed by the measurement of green fluorescence (excitation/emission=485/530nm) using a luminometer.

For colony formation assay, 500 cells were seeded in 6 well plates and allowed to form colonies. Colonies were maintained in the presence of vehicle control or CA3 (1 µM) or Verteporfin (2µM) for 72h. After 72h, colonies were fixed in ice-cold methanol for 5 min at -20ºC. Afterward, colonies were washed in PBS and stained with 0.5% crystal violet solution (made in 25% methanol). The colonies were scanned and counted using ImageJ software.

**Transfections and Dual-Luciferase Reporter (DLR) assay.**

PAF1 KD was performed in PC and KC cells by transfecting scramble or PAF1 siRNAs (PAF1 Human siRNA Oligo Duplex, Origene Cat#SR324271; or Paf1 mouse siRNA, Santacruz Cat#sc-76035) using Lipofectamine 3000 (Invitrogen Cat#L3000001) transfection reagent by following manufacturer’s instructions. The KD efficiency was tested using qRT-PCR and western blot. The role of PAF1 on the YAP/TEAD-mediated transcription and SOX9 promoter activity was monitored by an 8xTEAD-luciferase (8xGTIIC-luciferase) reporter and SOX9 promoter (pGL3-noSV40-mouseEC1.45) luciferase reporter assays, respectively. The 8xTEAD-luciferase reporter was obtained from Addgene (plasmid#34615), and pGL3-noSV40-mouseEC1.45 was purchased from Addgene (Plasmid#173972). Cells were plated in 24-well plates at 0.13 million cells per well and incubated overnight. Cells were transfected the next day with 500ng of 8xTEAD-luciferase or SOX9 reporter and 250 ng of Renilla luciferase reporter plasmid (pRL-CMV; Promega). For PAF1 KD experiments, 8xTEAD or SOX9 luciferase reporters, Renilla plasmid, and scramble or PAF1 siRNAs were co-transfected. All the transfections were performed using Lipofectamine 3000 (Invitrogen Cat#L3000001). For CA3 treatment experiments, cells were treated with vehicle control and CA3 for 48 h after transfections. At 48 h post-transfection, cells in 24 well plates were lysed in 100 µL of 1x passive lysis buffer. 20 µL of the lysate was added to the black 96 well plates (in triplicates), and the Luciferase activities were assayed using the Dual-luciferase reporter (DLR) assay system (Promega Cat#E1910) according to the manufacturer’s instructions. Firefly and Renilla luciferase activities were quantified using Synergy Neo2 multimode reader (BioTek, VT, USA), and the firefly luciferase activity was normalized for transfection efficiency using Renilla luciferase activity.

**IC50 (Half maximal inhibitory concentration) calculations**. Cells (0.01x10^6^) were seeded per well in 96-well plates. Cells were treated with different increasing concentrations of CA3 or Verteporfin. After 48h, Calcein AM staining was performed, followed by measuring the green fluorescence using Synergy Neo2 multimode reader (BioTek, VT, USA). IC50 values were calculated using an online IC50 calculator tool, AAT Bioquest. (https://www.aatbio.com/tools/ic50-calculator).

**Supplementary References**

1. Karmakar S, Rauth S, Nallasamy P, Perumal N, Nimmakayala RK, Leon F*, et al.* RNA Polymerase II-Associated Factor 1 Regulates Stem Cell Features of Pancreatic Cancer Cells, Independently of the PAF1 Complex, via Interactions With PHF5A and DDX3. *Gastroenterology* 2020, **159**(5)**:** 1898-1915.e1896.

2. Song S, Ajani JA, Honjo S, Maru DM, Chen Q, Scott AW*, et al.* Hippo coactivator YAP1 upregulates SOX9 and endows esophageal cancer cells with stem-like properties. *Cancer research* 2014, **74**(15)**:** 4170-4182.
