# Supplementary material for: PAF1 cooperates with YAP1 in metaplastic ducts to promote pancreatic cancer
Source: Cell Death Dis. 2022 Oct 1;13(10):839. doi: 10.1038/s41419-022-05258-x (PMC9525575; doi:10.1038/s41419-022-05258-x)
Supplement: Supplementary file 2 — Supplementary Fig1 [file 41419_2022_5258_MOESM2_ESM.pdf]

## Supplementary Figure 1

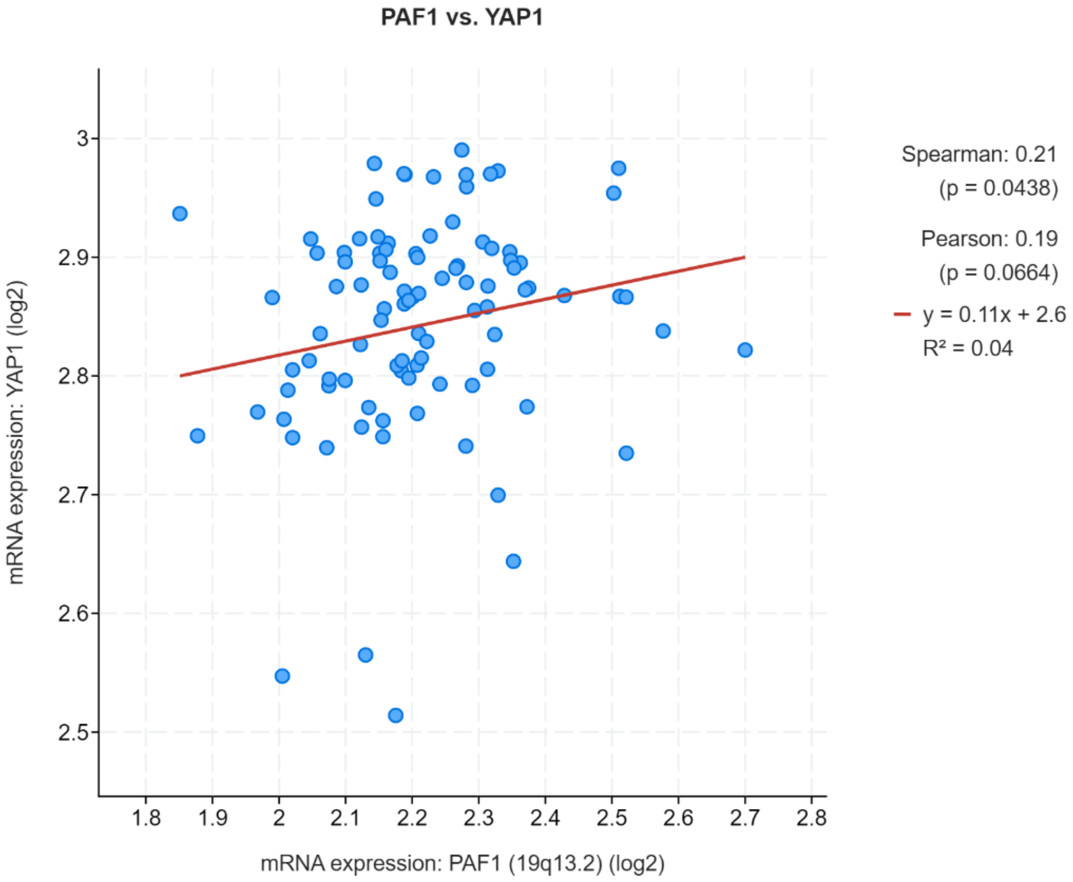


**Supplementary Figure 1. PAF1 expression is positively correlated with YAP1.** cBioportal analysis of the correlation of PAF1 with YAP1 in the Pancreatic Adenocarcinoma (QCMG, Nature 2016) dataset.
