# Supplementary material for: PAF1 cooperates with YAP1 in metaplastic ducts to promote pancreatic cancer
Source: Cell Death Dis. 2022 Oct 1;13(10):839. doi: 10.1038/s41419-022-05258-x (PMC9525575; doi:10.1038/s41419-022-05258-x)
Supplement: Supplementary file 3 — Supplementary Fig2 [file 41419_2022_5258_MOESM3_ESM.pdf]

Supplementary Figure 2

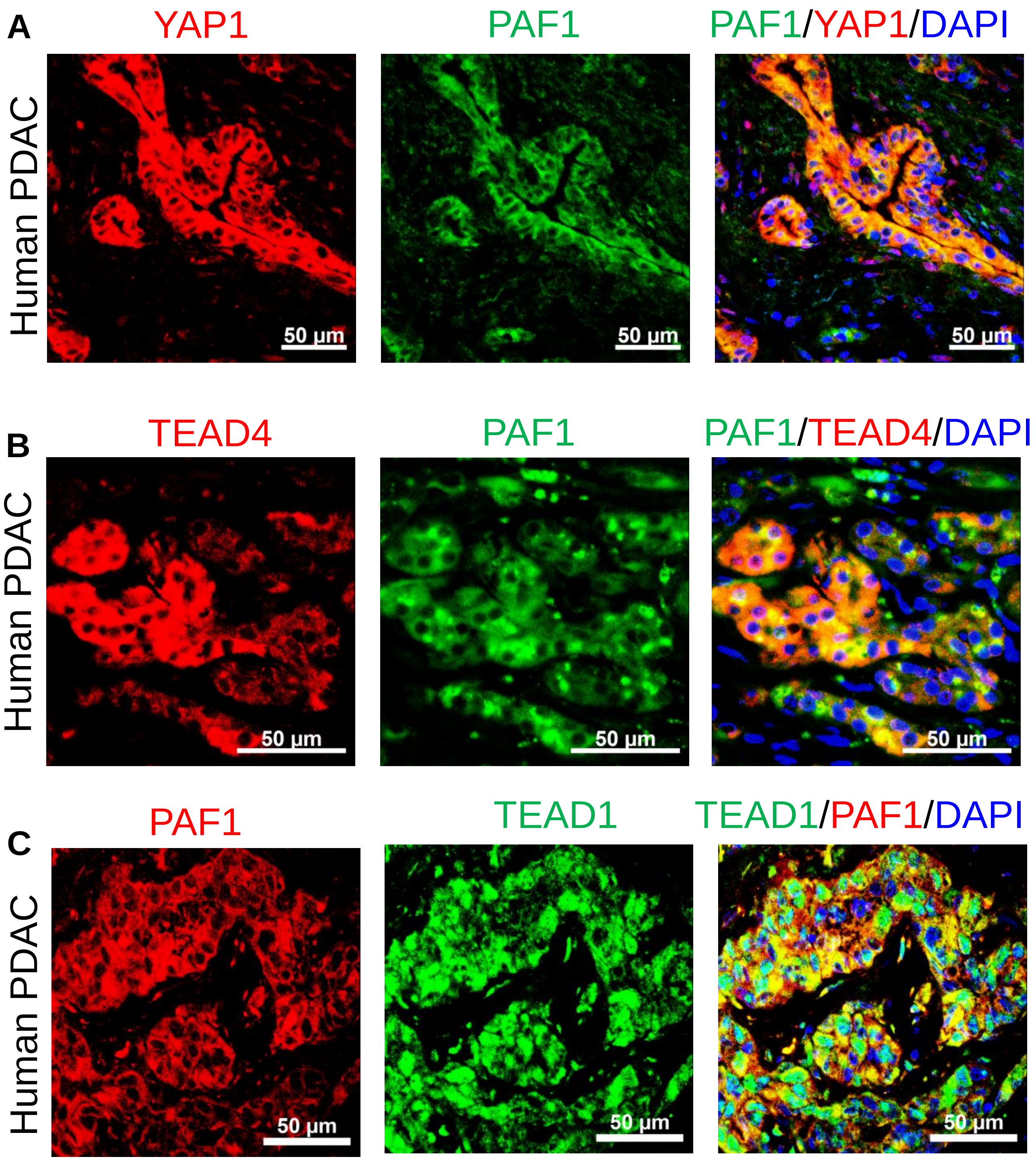

Supplementary Figure 2. Co-expression of PAF1 with YAP1, TEAD1, and TEAD4 in human PDAC. (**A, B, and C**), Immunofluorescence images of confocal microscopy.
