# Supplementary material for: PAF1 cooperates with YAP1 in metaplastic ducts to promote pancreatic cancer
Source: Cell Death Dis. 2022 Oct 1;13(10):839. doi: 10.1038/s41419-022-05258-x (PMC9525575; doi:10.1038/s41419-022-05258-x)
Supplement: Supplementary file 4 — Supplementary Fig3 [file 41419_2022_5258_MOESM4_ESM.pdf]

Supplementary Figure 3

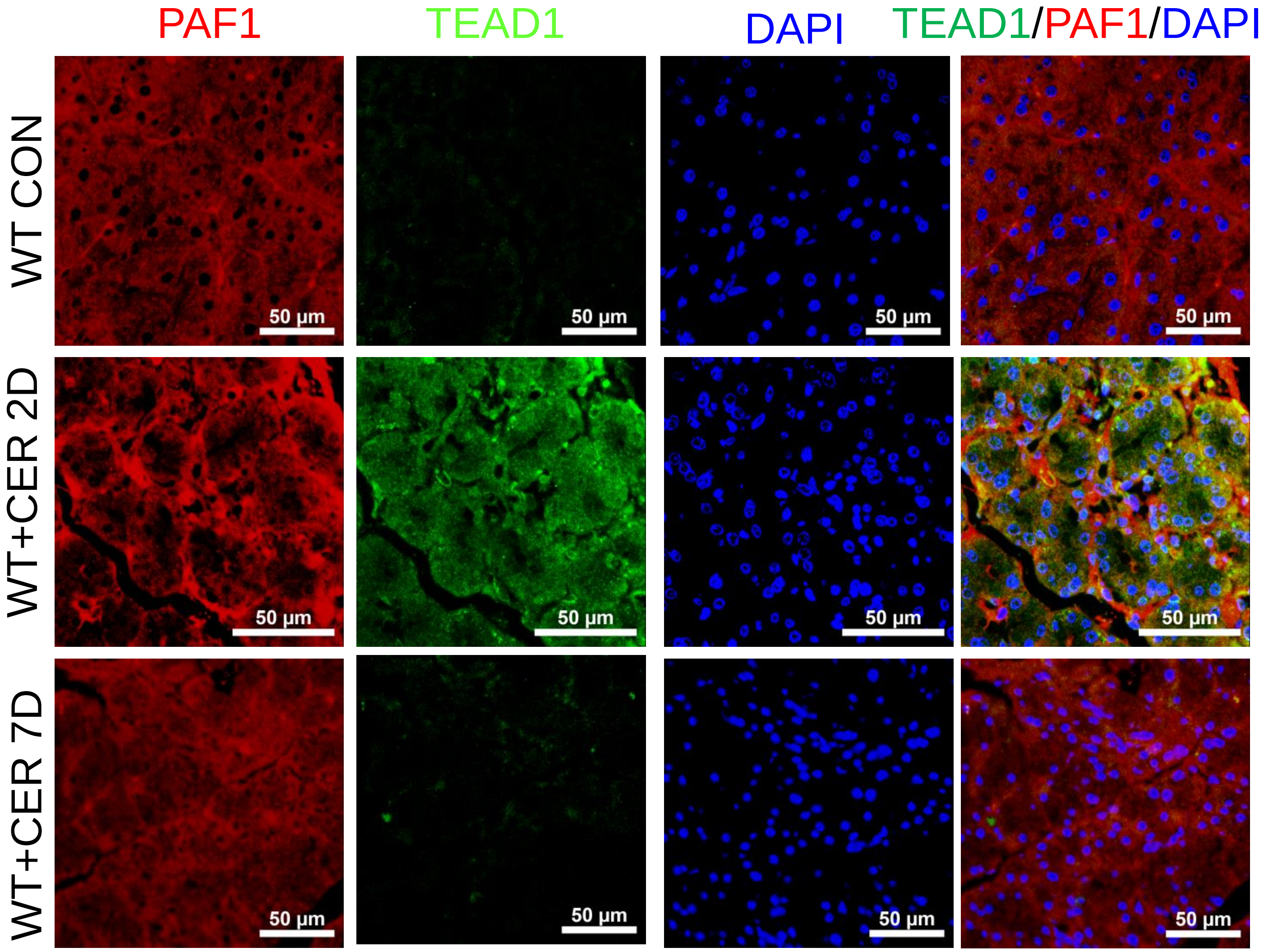

Supplementary Figure 3. Co-expression of PAF1 with TEAD1 in cerulein-induced wild-type (WT) acute pancreatitis mouse models. Immunofluorescence images of confocal microscopy.
