# Supplementary material for: PAF1 cooperates with YAP1 in metaplastic ducts to promote pancreatic cancer
Source: Cell Death Dis. 2022 Oct 1;13(10):839. doi: 10.1038/s41419-022-05258-x (PMC9525575; doi:10.1038/s41419-022-05258-x)
Supplement: Supplementary file 5 — Supplementary Fig4 [file 41419_2022_5258_MOESM5_ESM.pdf]

Supplementary Figure 4

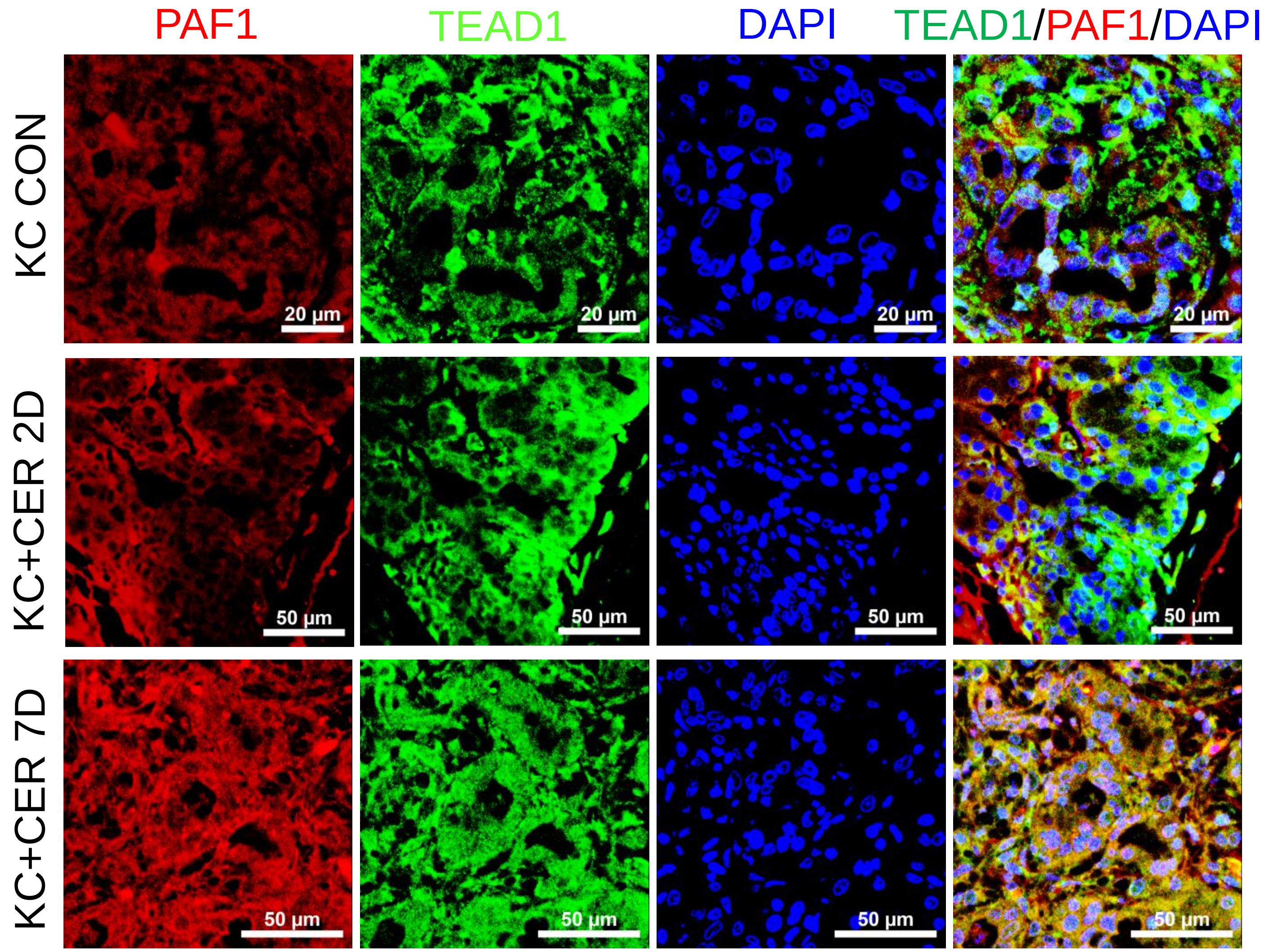

Supplementary Figure 4. Co-expression of PAF1 with TEAD1 in cerulein-induced Kras^{G12D}; Pdx1 Cre (KC) acute pancreatitis mouse models. Immunofluorescence images of confocal microscopy.
