# Supplementary material for: PAF1 cooperates with YAP1 in metaplastic ducts to promote pancreatic cancer
Source: Cell Death Dis. 2022 Oct 1;13(10):839. doi: 10.1038/s41419-022-05258-x (PMC9525575; doi:10.1038/s41419-022-05258-x)
Supplement: Supplementary file 6 — Supplementary Fig5 [file 41419_2022_5258_MOESM6_ESM.pdf]

Supplementary Figure 5

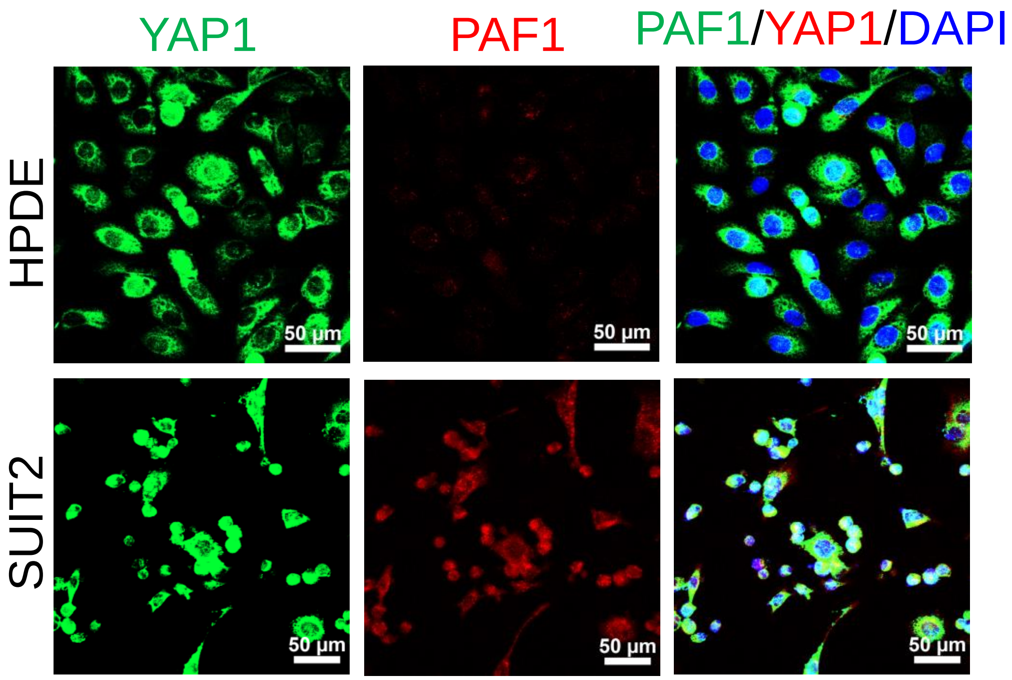

Supplementary Figure 5. Co-localization of PAF1 and YAP1 in PC cells (SUIT2) but not in human pancreatic normal ductal epithelial (HPDE) cells. Confocal images of Immunofluorescence staining for PAF1 and YAP1 in indicated cell lines.
