# Supplementary material for: PAF1 cooperates with YAP1 in metaplastic ducts to promote pancreatic cancer
Source: Cell Death Dis. 2022 Oct 1;13(10):839. doi: 10.1038/s41419-022-05258-x (PMC9525575; doi:10.1038/s41419-022-05258-x)
Supplement: Supplementary file 7 — Supplementary Fig6 [file 41419_2022_5258_MOESM7_ESM.pdf]

Supplementary Figure 6

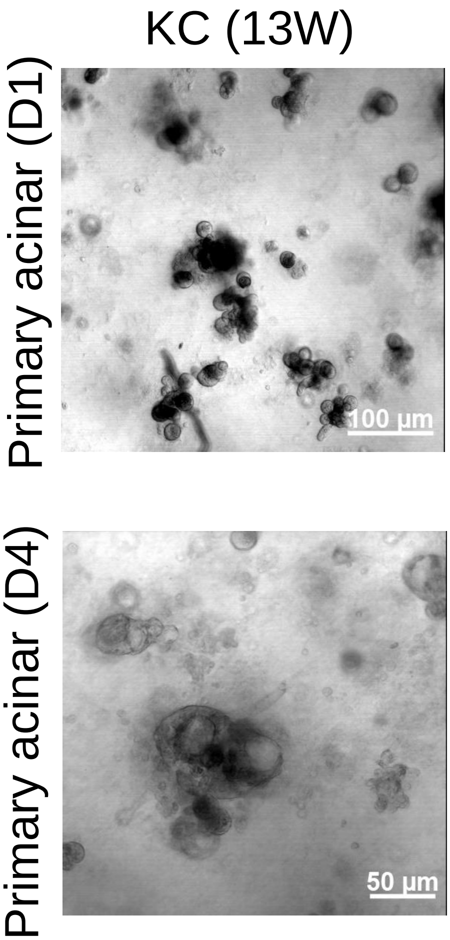

Supplementary Figure 6. Light microscope images of primary pancreatic acinar cells. Isolated pancreatic acini from KrasG12D; Pdx-1 Cre (KC) (13 weeks) mice were cultured in collage under 3D conditions (acinar explants embedded in collagen). Images were captured on day 1 and 4.
