# Supplementary material for: PAF1 cooperates with YAP1 in metaplastic ducts to promote pancreatic cancer
Source: Cell Death Dis. 2022 Oct 1;13(10):839. doi: 10.1038/s41419-022-05258-x (PMC9525575; doi:10.1038/s41419-022-05258-x)
Supplement: Supplementary file 8 — Supplementary Fig7 [file 41419_2022_5258_MOESM8_ESM.pdf]

Supplementary Figure 7

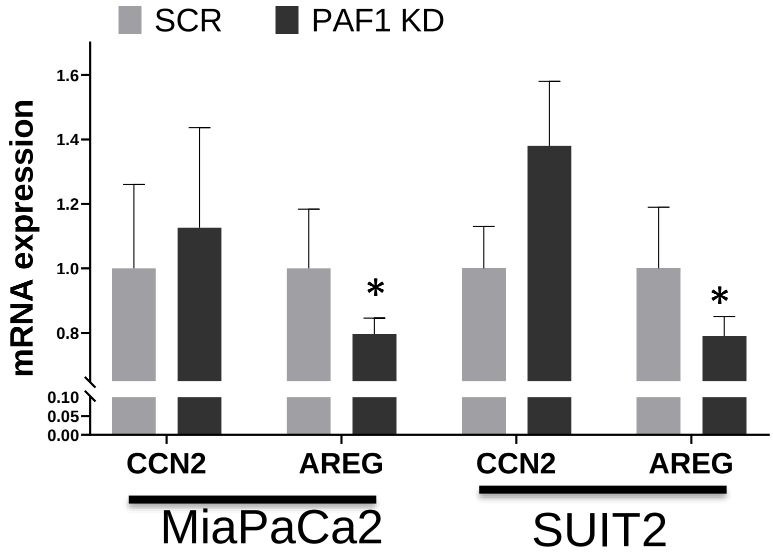

Supplementary Figure 7. PAF1 KD reduces the expression of YAP1 target gene, AREG. QPCR analysis of YAP1 target genes, AREG and CCN2 in MiaPaCa2 and SUIT2 pancreatic cancer cells. qRT-PCR data were normalized with the Actb gene. Data are mean ±S.D., n = 3. Significance was determined with a student's t-test. *p<0.05.
