# Supplementary material for: PAF1 cooperates with YAP1 in metaplastic ducts to promote pancreatic cancer
Source: Cell Death Dis. 2022 Oct 1;13(10):839. doi: 10.1038/s41419-022-05258-x (PMC9525575; doi:10.1038/s41419-022-05258-x)
Supplement: Supplementary file 9 — Supplementary Fig8 [file 41419_2022_5258_MOESM9_ESM.pdf]

Supplementary Figure 8

A IC50 value of CA3 for 9-26 NP cells: **1612** nM

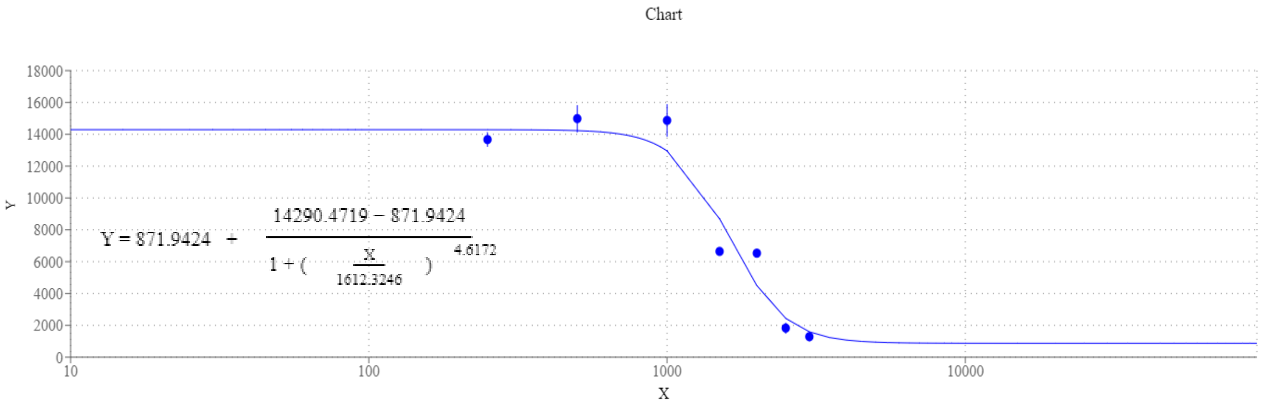

B IC50 value of CA3 for SUIT2 cells: **1097** nM

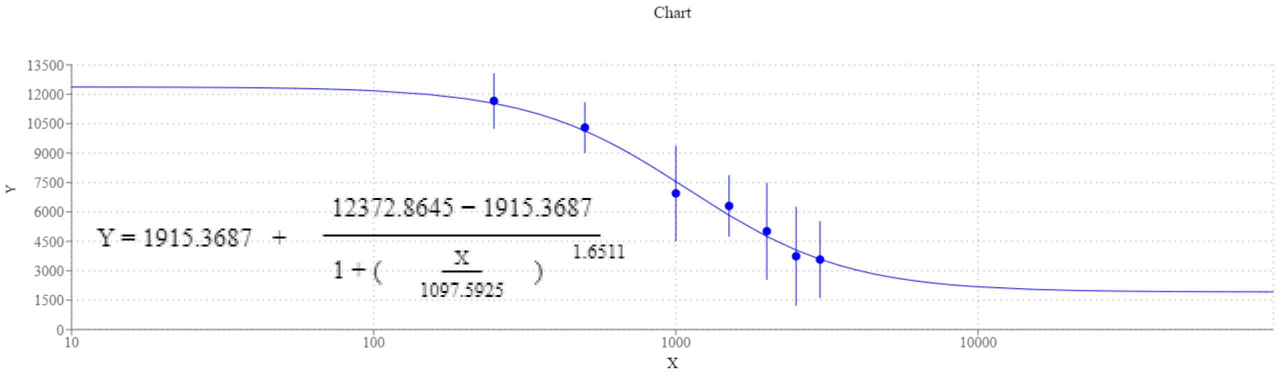

Supplementary Figure 8. IC50 value for normal human fibroblast cell line is higher than PC cell line. A and B, 9-26 NP (normal human fibroblast cell line) and SUIT2 (PC cell line) were treated with CA3 (at increasing concentrations) for 48 hr. Calcein-AM assay was used to stain the viable cells.
