# Supplementary material for: PAF1 cooperates with YAP1 in metaplastic ducts to promote pancreatic cancer
Source: Cell Death Dis. 2022 Oct 1;13(10):839. doi: 10.1038/s41419-022-05258-x (PMC9525575; doi:10.1038/s41419-022-05258-x)
Supplement: Supplementary file 10 — Supplementary Fig9 [file 41419_2022_5258_MOESM10_ESM.pdf]

Supplementary Figure 9

IC50 value of CA3 for MiaPaCa2 cells: **913.49** nM

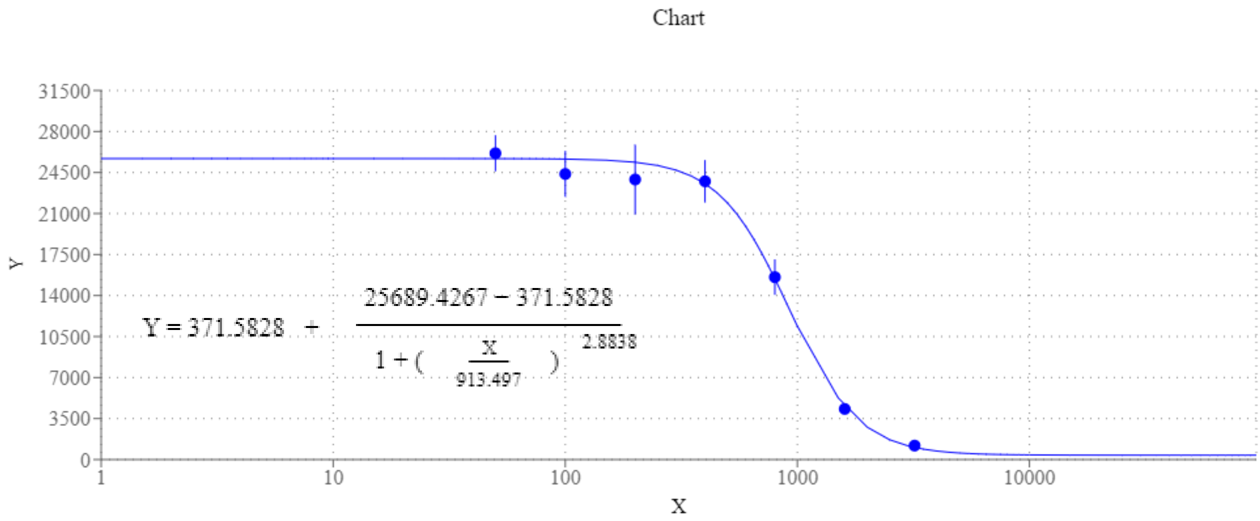

Supplementary Figure 9. IC50 value of CA3 for MiaPaCa2 PC cell line. MiaPaCa2 PC cells were treated with CA3 (at increasing concentrations) for 48 hr. Calcein-AM assay was used to stain the viable cells.
