# Supplementary material for: PAF1 cooperates with YAP1 in metaplastic ducts to promote pancreatic cancer
Source: Cell Death Dis. 2022 Oct 1;13(10):839. doi: 10.1038/s41419-022-05258-x (PMC9525575; doi:10.1038/s41419-022-05258-x)
Supplement: Supplementary file 11 — Supplementary Fig10 [file 41419_2022_5258_MOESM11_ESM.pdf]

Supplementary Figure 10

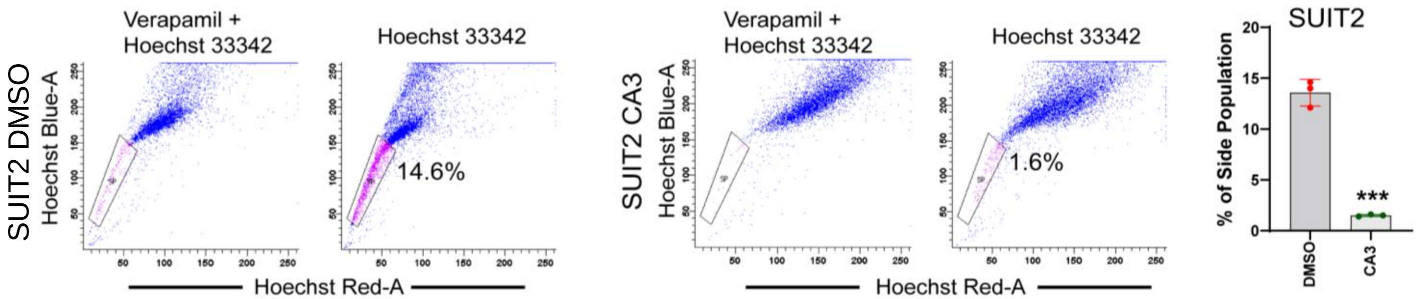

Supplementary Figure 10. CA3 inhibits side population (SP) in PC cells. Flow cytometry analysis of side population (SP) or drug-resistant population in vehicle control or CA3 treated PC cells. The bar graph shows the percentage of the SP population. Data are mean ±S.D., n = 3. Significance was determined with a student's t-test. *p<0.05, **p<0.01, ***p<0.001, ns: non-significant, p>0.05.
