# Supplementary material for: PAF1 cooperates with YAP1 in metaplastic ducts to promote pancreatic cancer
Source: Cell Death Dis. 2022 Oct 1;13(10):839. doi: 10.1038/s41419-022-05258-x (PMC9525575; doi:10.1038/s41419-022-05258-x)
Supplement: Supplementary file 12 — Supplementary Fig11 [file 41419_2022_5258_MOESM12_ESM.pdf]

Supplementary Figure 11

IC50 value of Verteporfin for MiaPaCa2 cells: **2182.47** nM

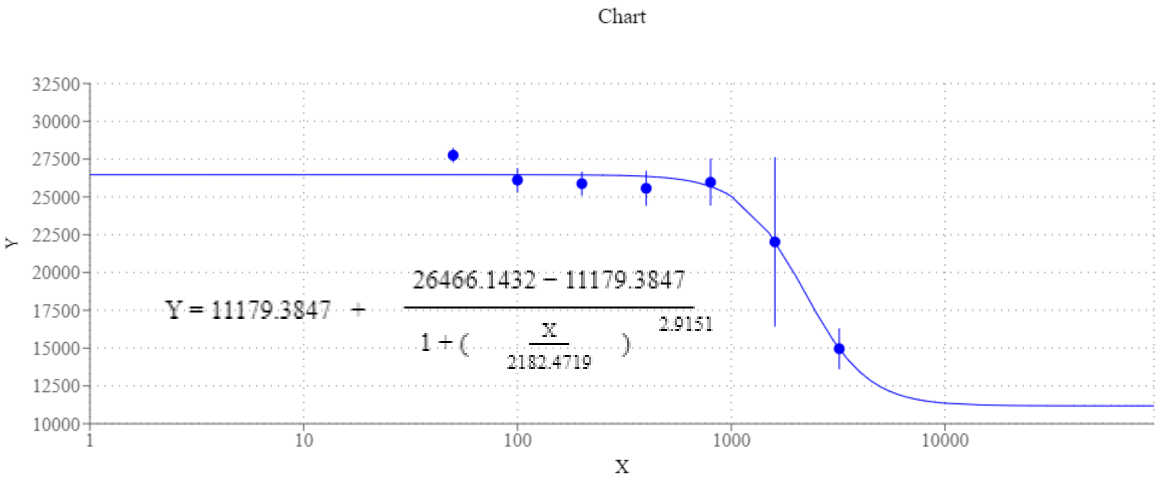

Supplementary Figure 11. IC50 value of Verteporfin for MiaPaCa2 PC cell line. MiaPaCa2 PC cells were treated with Verteporfin (at increasing concentrations) for 48 hr. Calcein-AM assay was used to stain the viable cells.
