# Supplementary material for: PAF1 cooperates with YAP1 in metaplastic ducts to promote pancreatic cancer
Source: Cell Death Dis. 2022 Oct 1;13(10):839. doi: 10.1038/s41419-022-05258-x (PMC9525575; doi:10.1038/s41419-022-05258-x)
Supplement: Supplementary file 13 — Supplementary Fig12 [file 41419_2022_5258_MOESM13_ESM.pdf]

Supplementary Figure 12

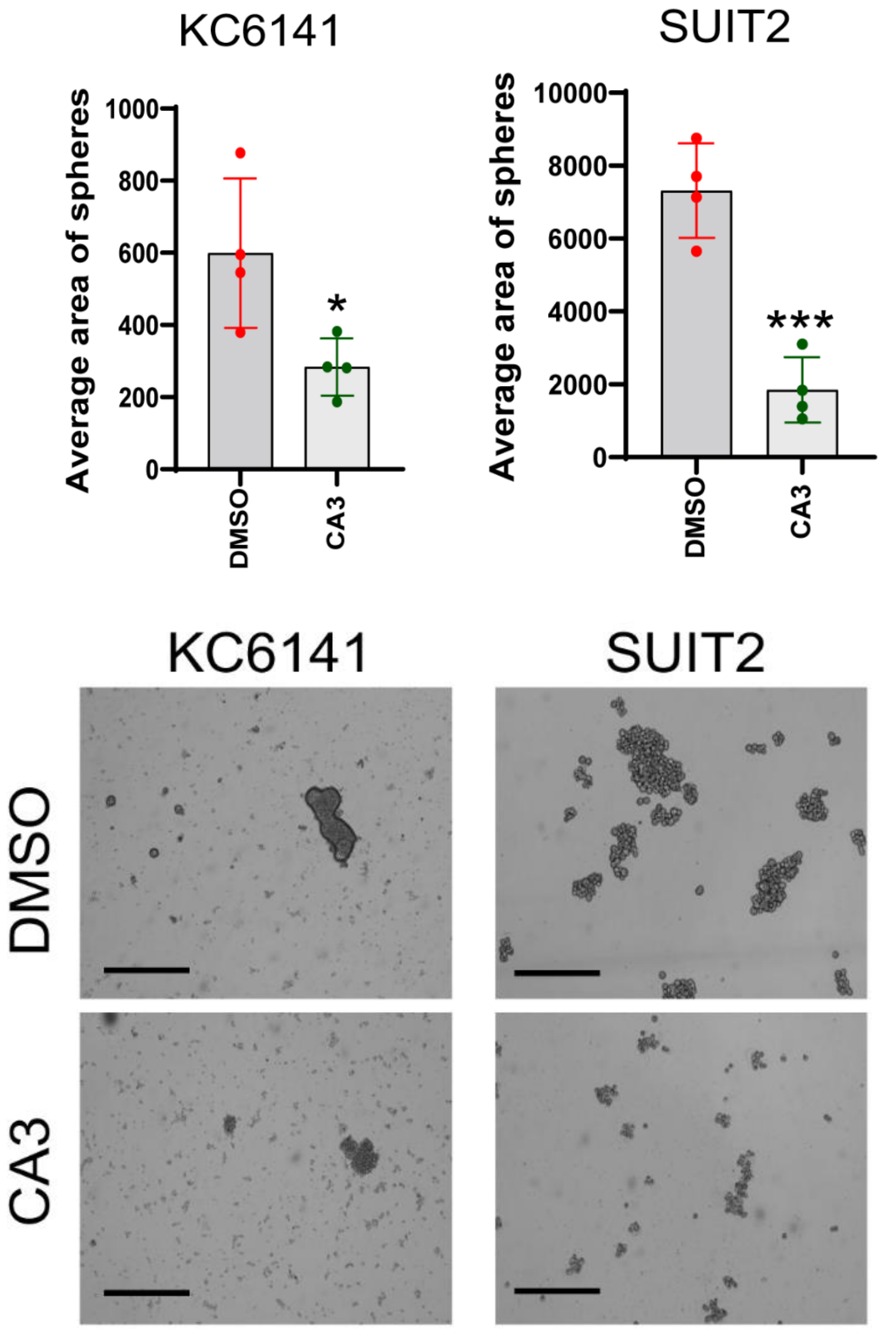

Supplementary Figure 12. Tumorsphere assay. SUIT2 or KC6141 cells were seeded in ultra-low attachment plates in a stem cell-specific medium in the presence or absence of CA3. Incucyte live cell imaging system was used to monitor the sphere formation. Bar graphs show the average area of spheres. For all panels, significance was determined with a student's t-test. *p<0.05, **p<0.01, ***p<0.001, ns: non-significant, p>0.05.
