# Supplementary material for: PAF1 cooperates with YAP1 in metaplastic ducts to promote pancreatic cancer
Source: Cell Death Dis. 2022 Oct 1;13(10):839. doi: 10.1038/s41419-022-05258-x (PMC9525575; doi:10.1038/s41419-022-05258-x)
Supplement: Supplementary file 14 — Supplementary Fig13 [file 41419_2022_5258_MOESM14_ESM.pdf]

Supplementary Figure 13

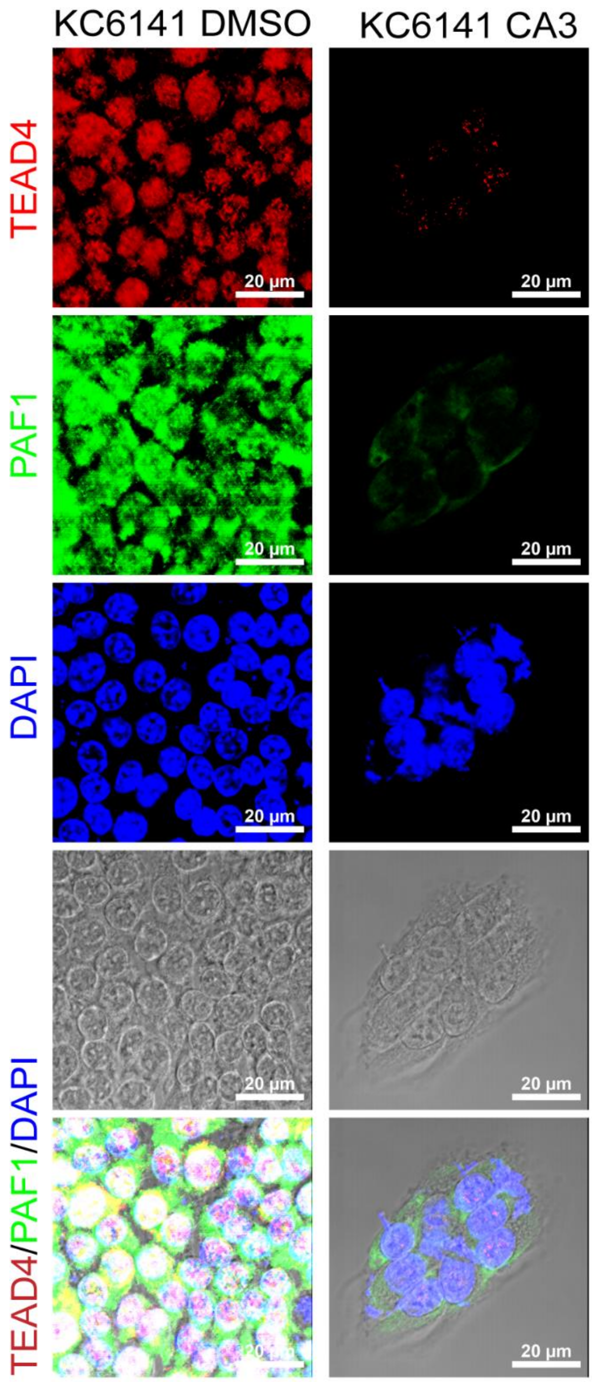

Supplementary Figure 13. Co-localization of PAF1 and TEAD4 in PC cells. KC6141 cells were treated with vehicle or CA3 (1µM) for 48hr. Confocal images of Immunofluorescence staining for PAF1 and YAP1 in indicated cells with indicated treatments.
