# Supplementary material for: PAF1 cooperates with YAP1 in metaplastic ducts to promote pancreatic cancer
Source: Cell Death Dis. 2022 Oct 1;13(10):839. doi: 10.1038/s41419-022-05258-x (PMC9525575; doi:10.1038/s41419-022-05258-x)

Figure. 3C full blots

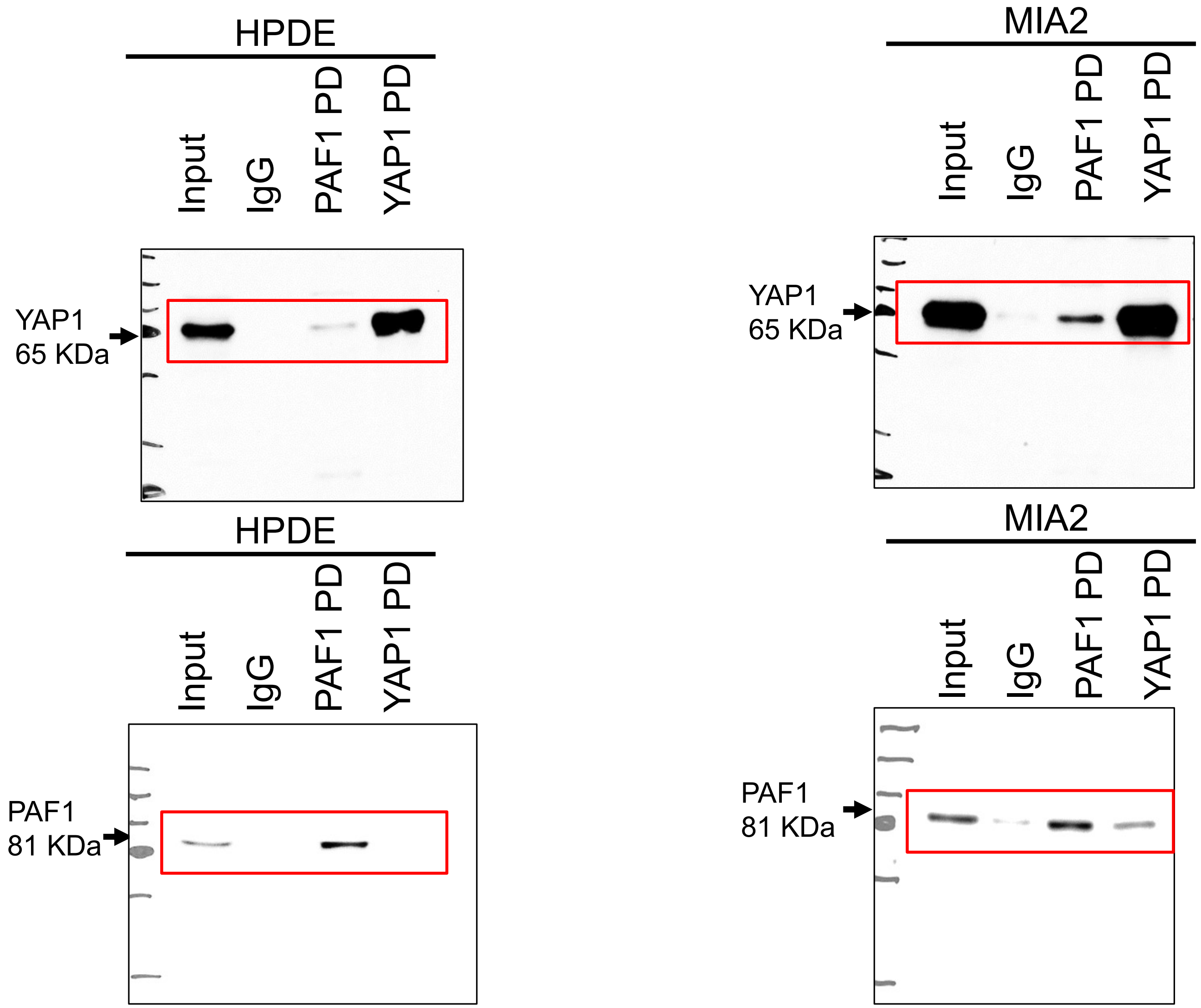

Figure. 3D full blots

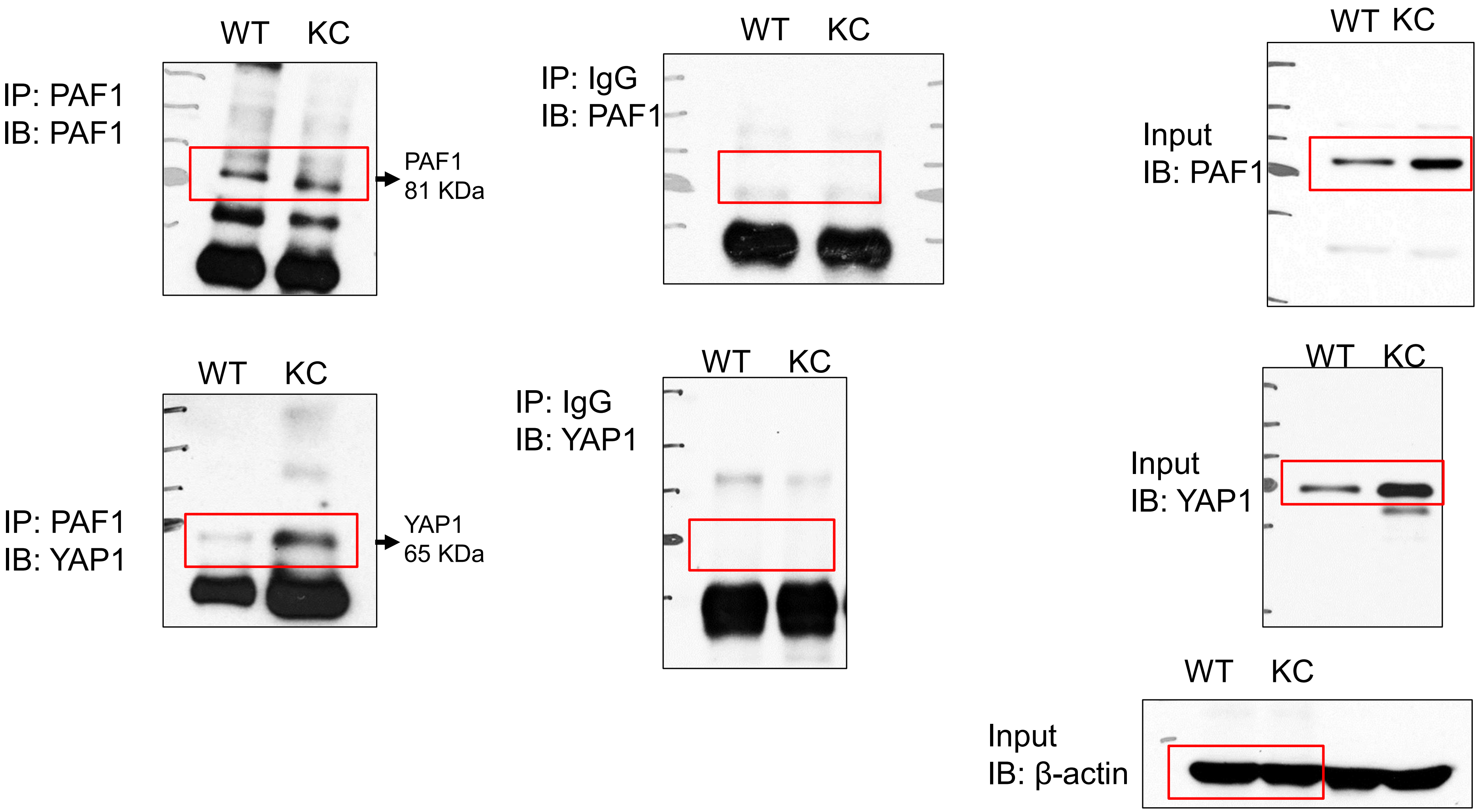

Figure. 3E full blots

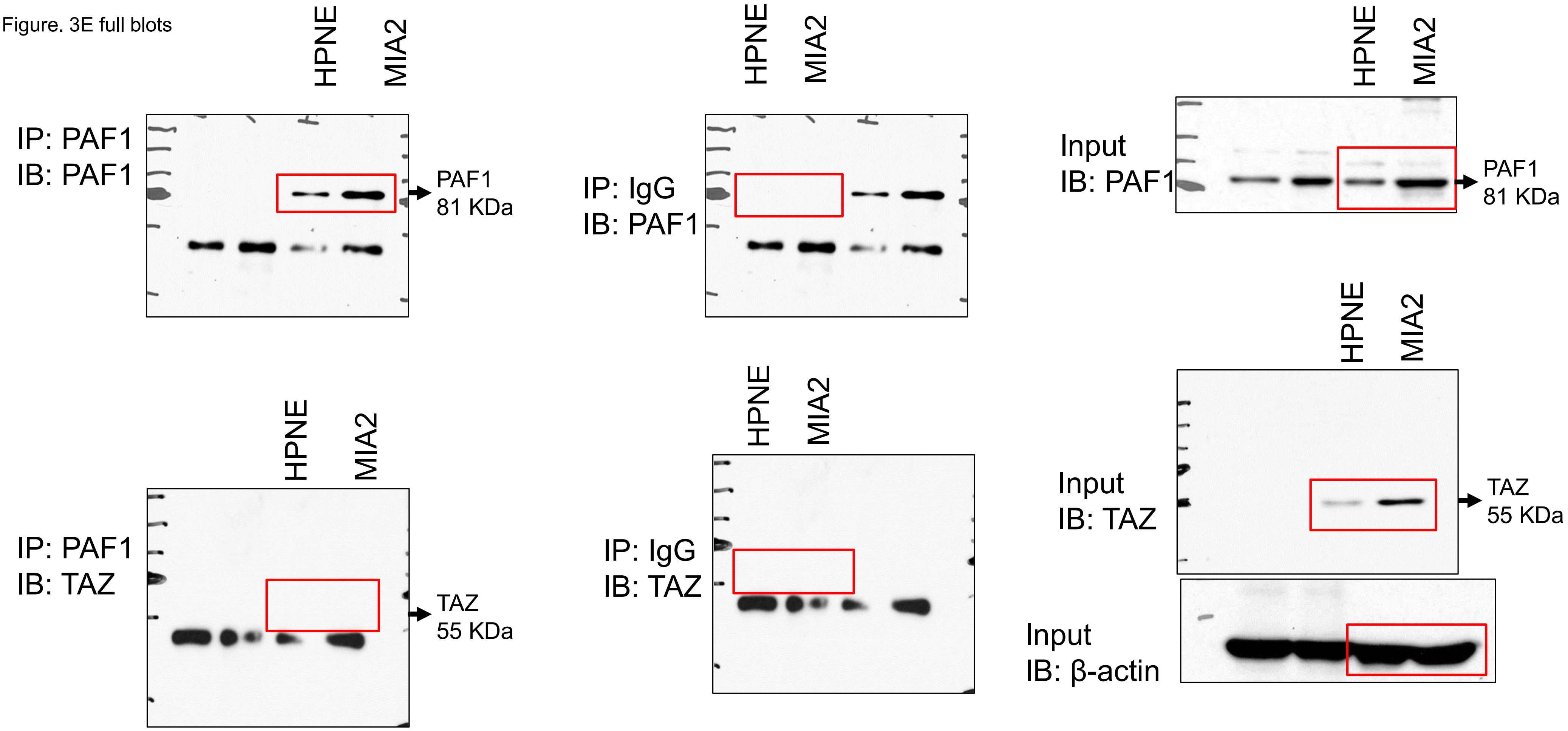

Figure. 3H full blots

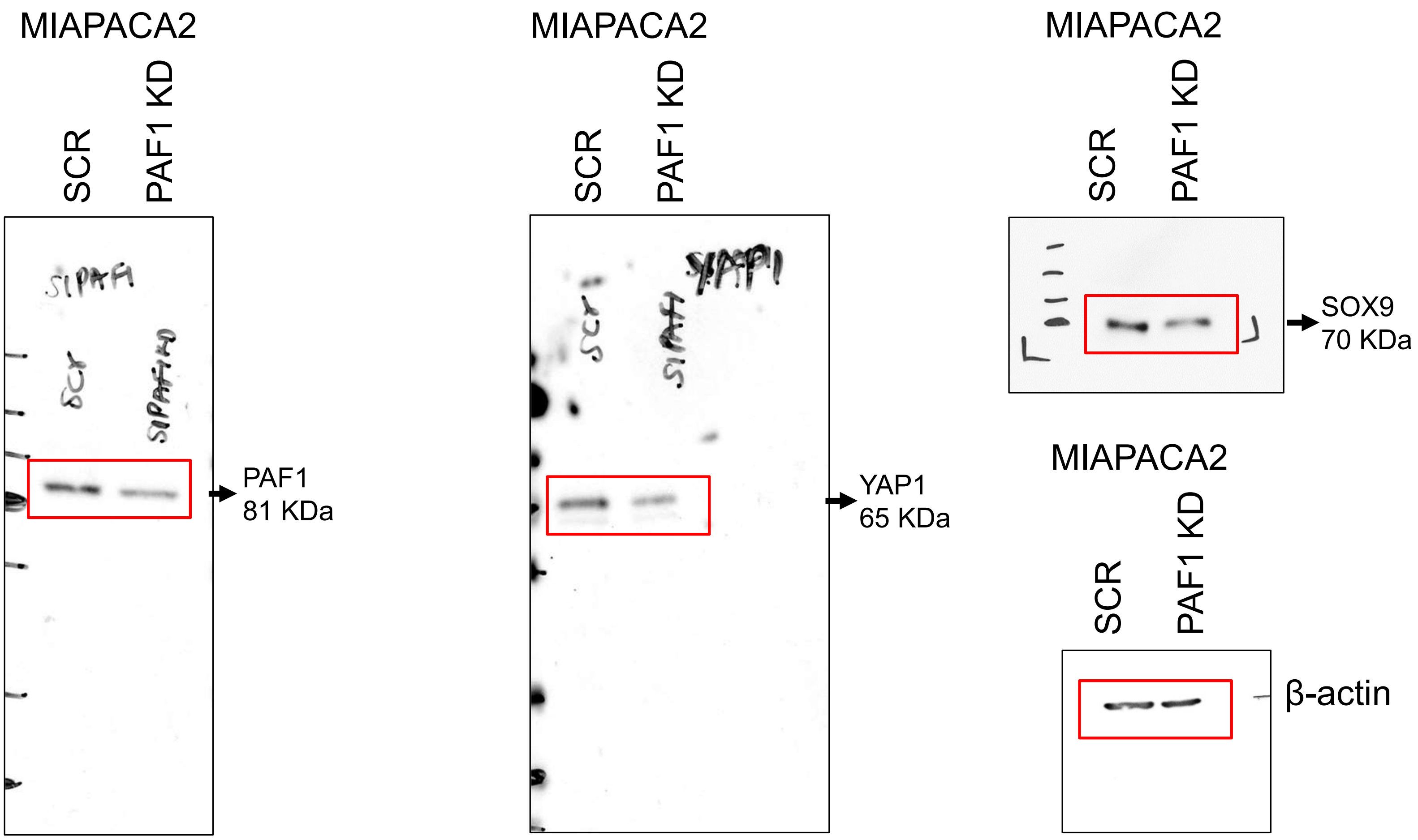

Figure. 4A full blots

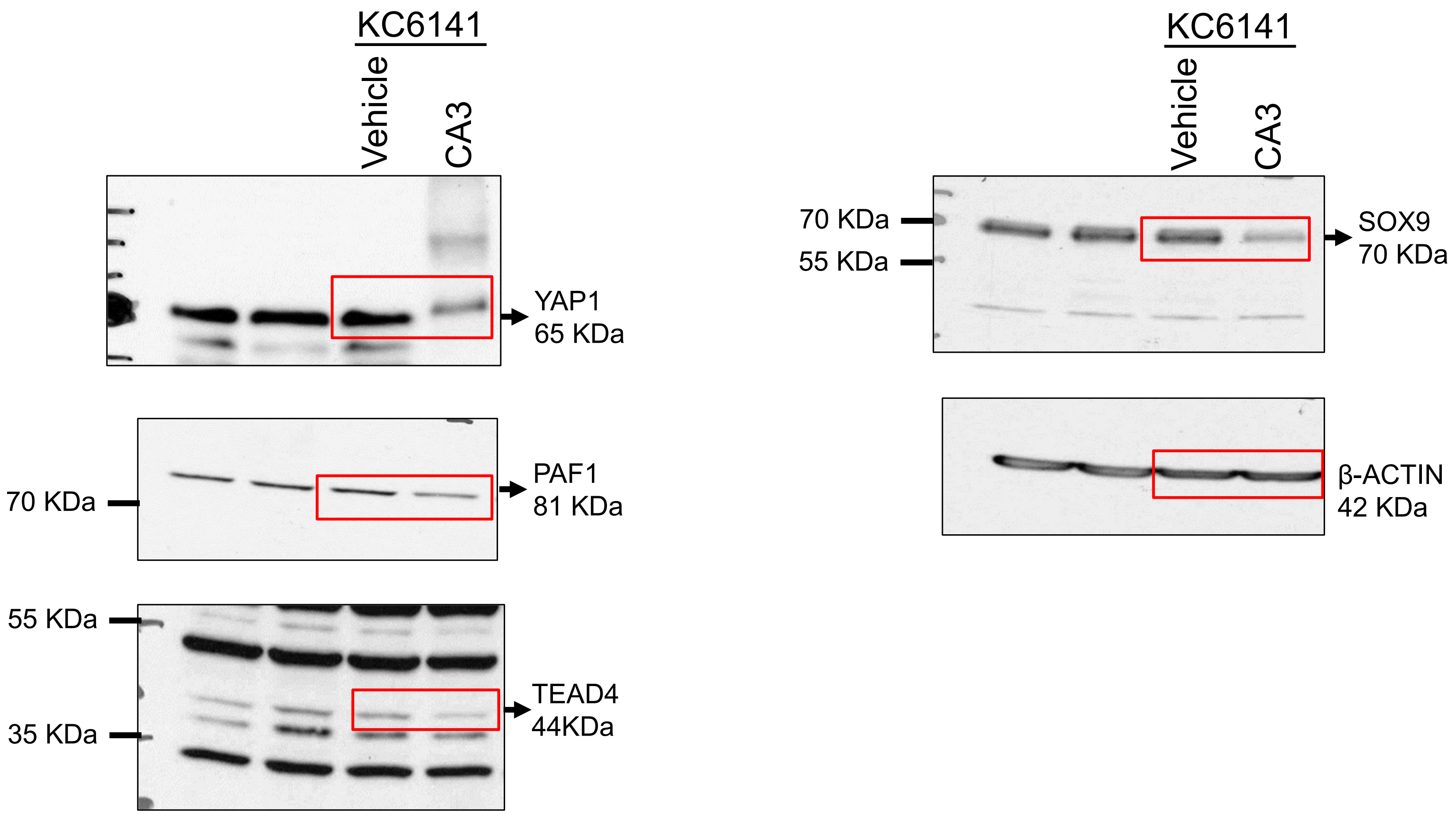

Figure. 4B full blots

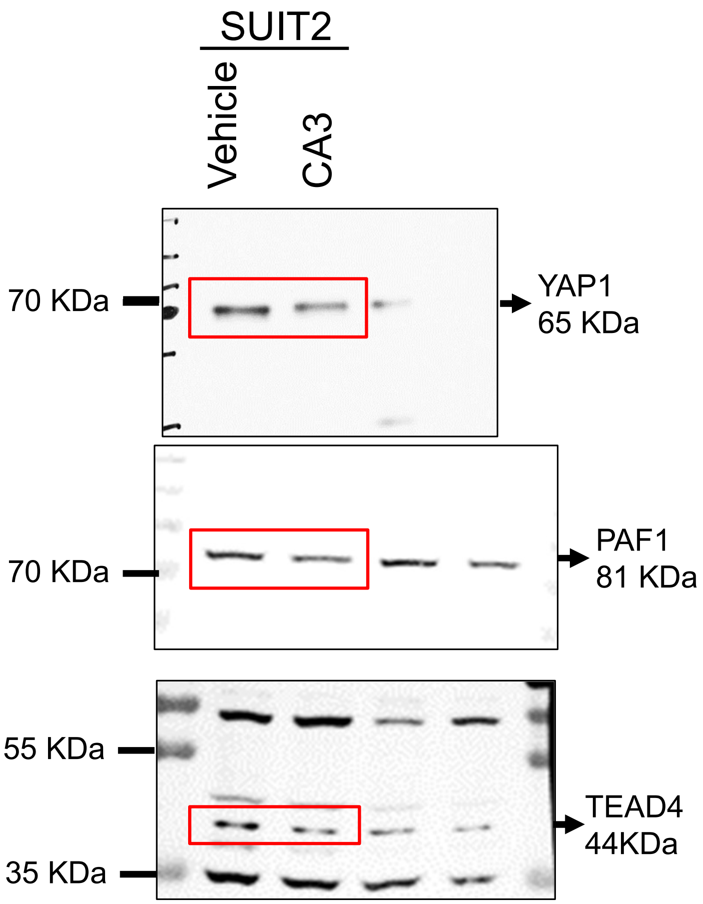

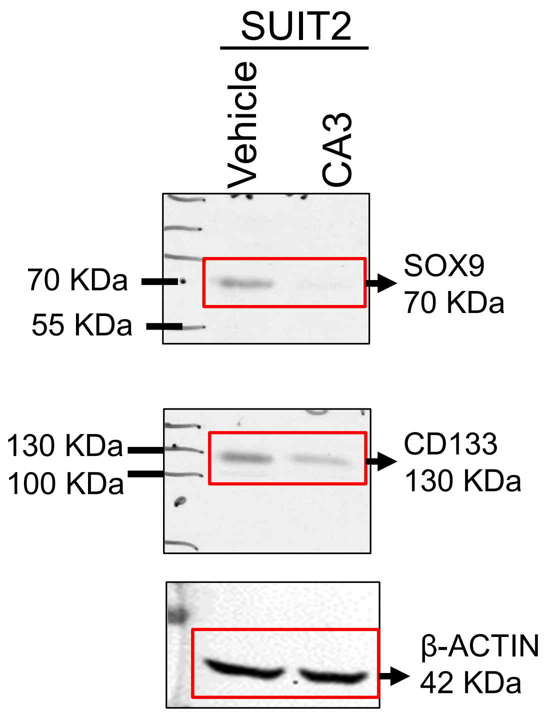

Figure. 4C full blots

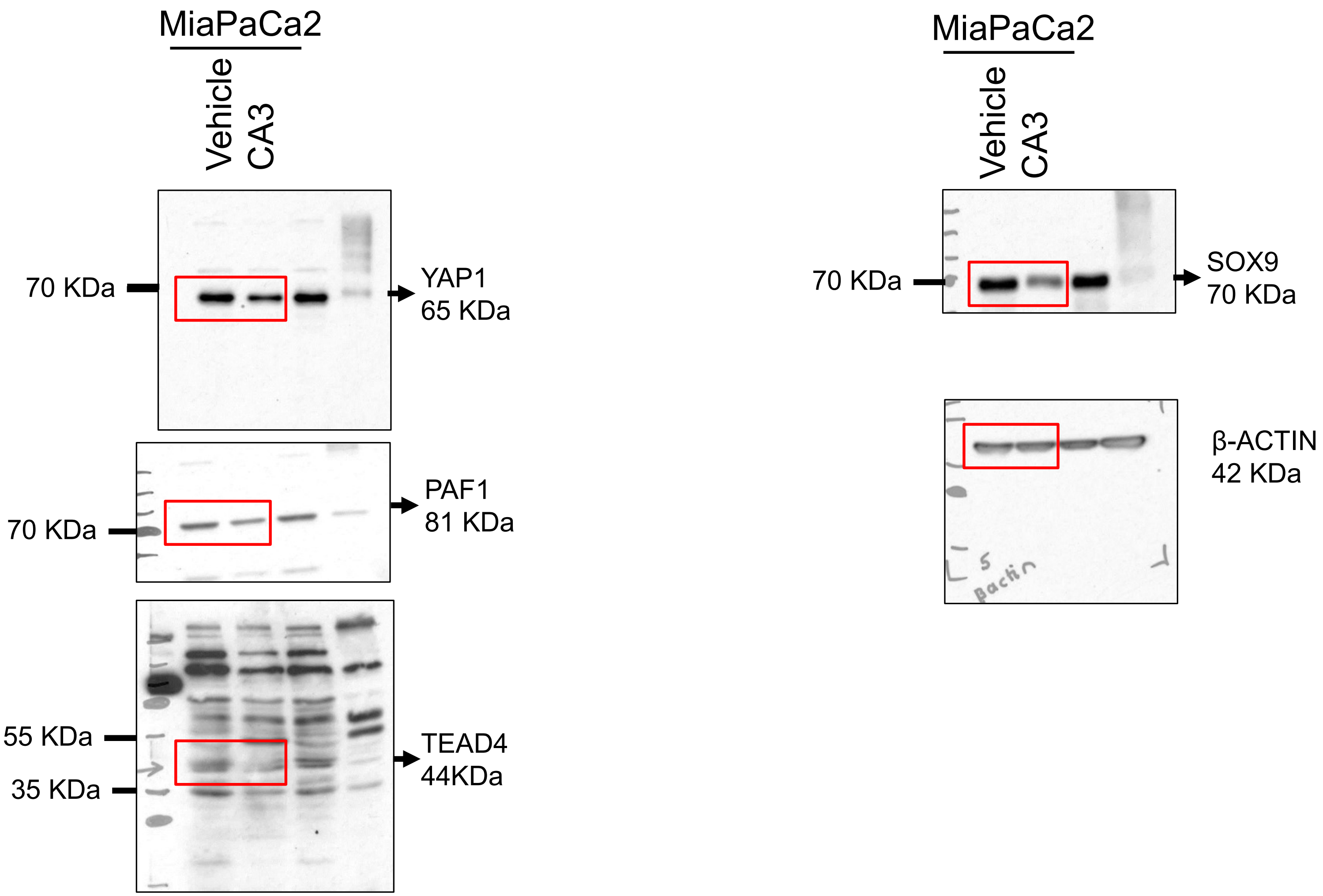

Figure. 4F full blots

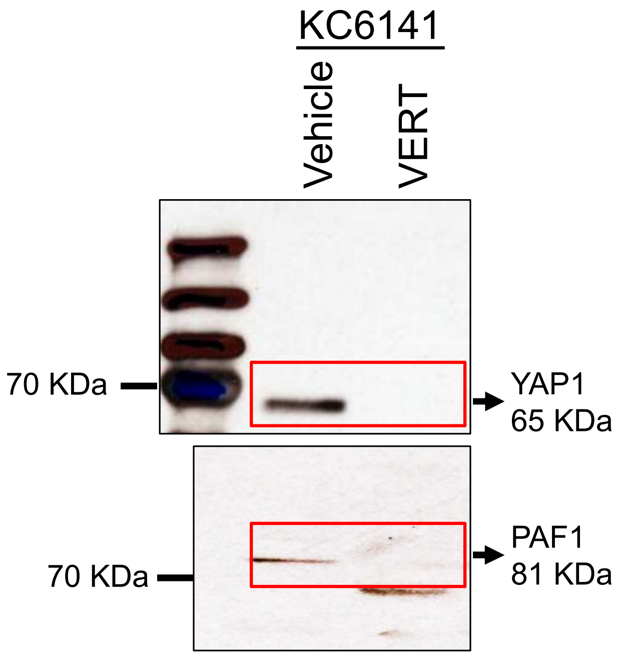

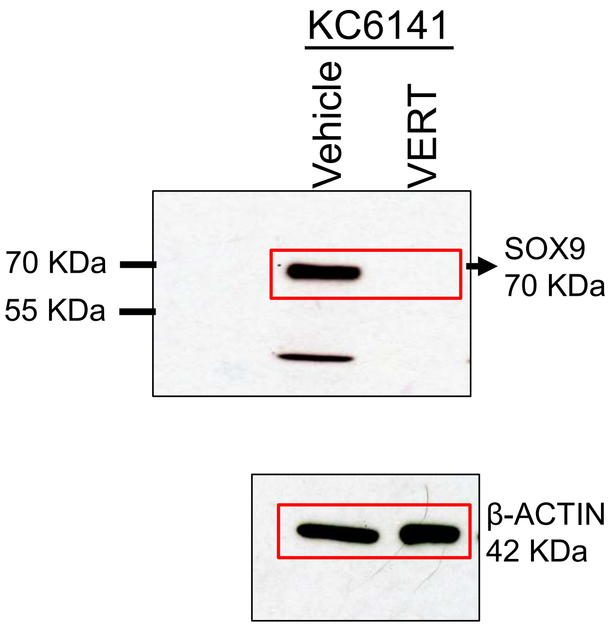

Figure. 4G full blots

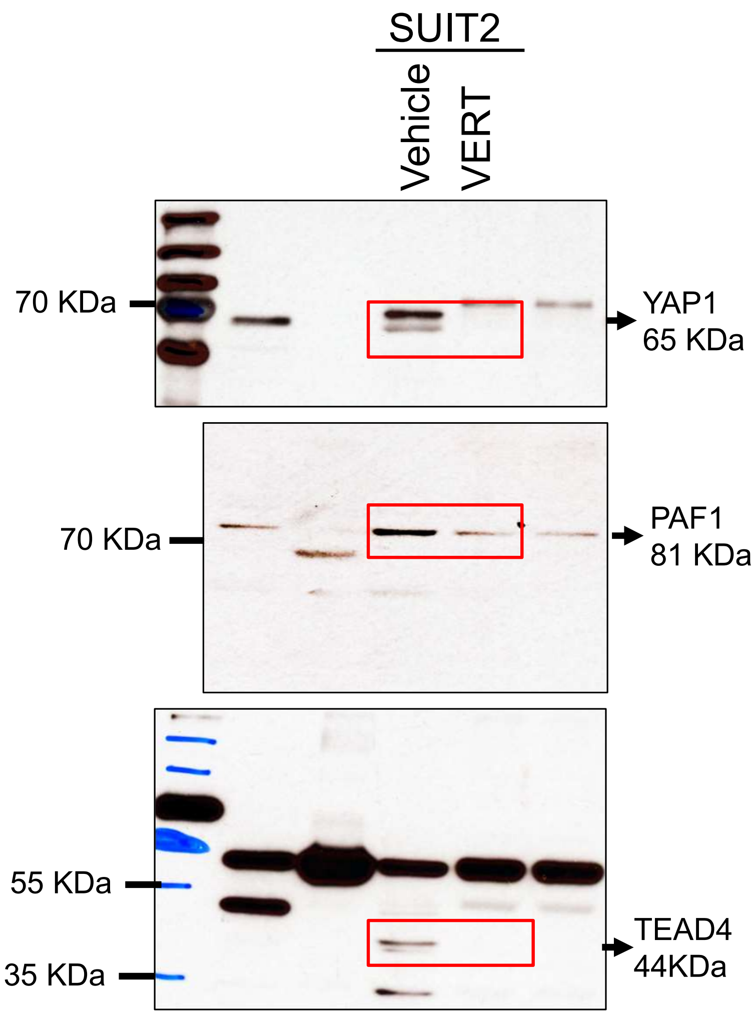

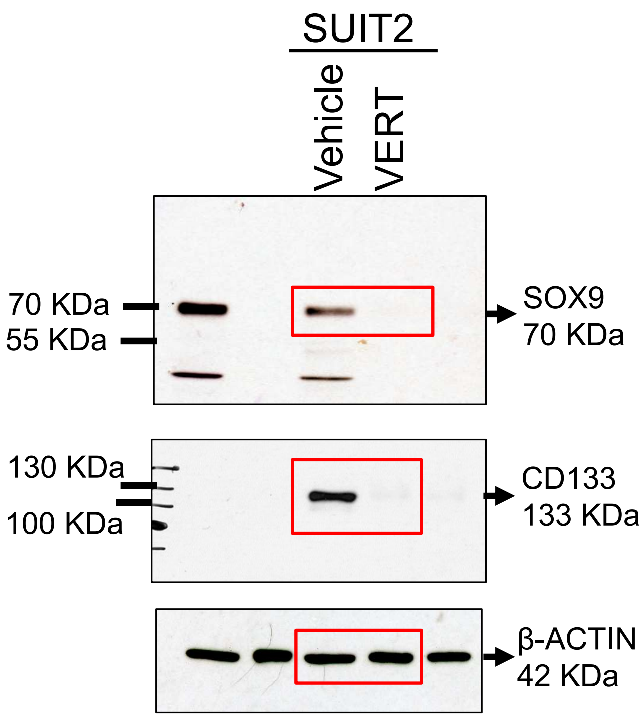

Figure. 4H full blots

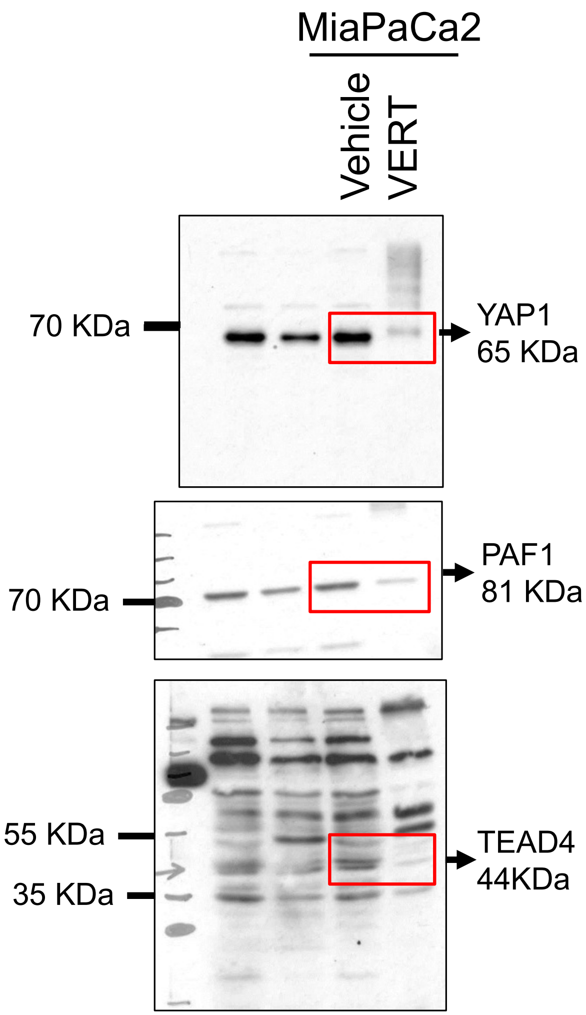

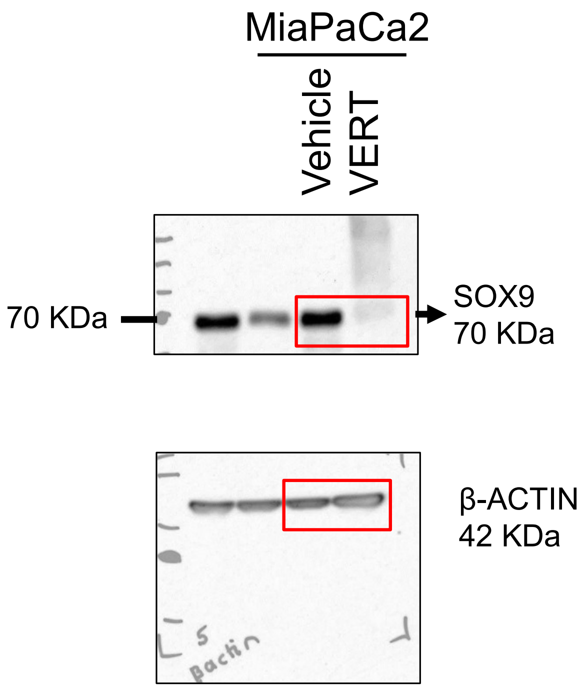

Supplement: Supplementary file 15 — Original Data File [file 41419_2022_5258_MOESM15_ESM.pdf]
